# Learning reorganizes dendritic and stabilizes axon initial segment inhibitory synapses in CA1 pyramidal neurons

**DOI:** 10.1038/s41467-026-77800-w

**Published:** 2026-09-14

**Authors:** Hannah Klimmt, Felix Kuhn, David Kappel, Bhargavi Murthy, Alessandro Francesco Ulivi, Ali Öztürk Argunşah, Silvia Vieweg, Rosa Eva Huettl, Stefan Remy, Alessio Attardo

**Affiliations:** 1 Leibniz Institute for Neurobiology, Magdeburg, Germany; 2 International Max Planck Research School for Translational Psychiatry, Munich, Germany; 3 Center for Cognitive Interaction Technology, Bielefeld University, Bielefeld, Germany; 4 Graduate School for Systemic Neuroscience, Ludwig-Maximilian University, Munich, Germany; 5 Brain Research Institute (HiFo), University of Zurich and Neuroscience Center Zurich, Zurich, Switzerland; 6 Max Planck Institute of Psychiatry, Munich, Germany; 7 German Center for Mental Health (DZPG), partner site Halle-Jena-Magdeburg and Center for Behavioral Brain Science (CBBS), Magdeburg, Germany; 8 German Center for Neurodegenerative Diseases (DZNE), Magdeburg, Germany

**Keywords:** Fear conditioning, Cellular neuroscience, Synaptic plasticity

## Abstract

Structural synaptic plasticity underlies the changes in brain connectivity required for learning and memory. Inhibitory synapses target all subcellular domains of excitatory pyramidal neurons, including dendrites, somata and axon initial segments. These subcellular domains have distinct molecular, structural and physiological profiles which underlie their functions. How structural plasticity of inhibitory synapses supports these functions as well as emerging properties such as memory is largely unknown. To tackle these questions we tracked inhibitory synapses on basal dendrites, somata and axon initial segments of pyramidal neurons in the dorsal hippocampal CA1 area of mice over two weeks. Size and temporal dynamics of inhibitory synapses showed a strong compartmentalization. Trace fear conditioning led to reorganization of dendritic and to stabilization of axon initial segments’ inhibitory synapses. Finally, mathematical modelling allowed us to probe the mechanisms underlying stabilization of inhibitory synapses upon learning.

## Abstract (web summary)

Using repeated brain imaging, the authors find that inhibitory synapses change at different rates across neuronal subcellular compartments in mouse CA1. Fear learning reorganizes dendritic connections but stabilizes those at the axon initial segment, which may aid memory recall.

## Introduction

Coordinated changes in synaptic connections give rise to the brain’s ability to learn and recall information^1–3^. These modifications include strengthening or weakening of existing synapses, as well as synapse formation and elimination. Structural synaptic plasticity underlies long-term changes in connectivity that might be required for learning and memory^4,5^. Moreover, the interplay between excitatory and inhibitory synaptic transmission serves an important role in adult brain and plasticity of excitatory and inhibitory inputs both participate in the processing and integration of local dendritic activity^6,7^.

Inhibitory synapses (ISs) target all subcellular domains of excitatory PNs (PNs), including the dendritic arborizations, the soma, and the axon initial segment (AIS)^8,9^. These subcellular domains have distinct molecular, structural, and physiological profiles which underlie their specific computational functions: dendrites receive and integrate inputs, while somata and AIS provide the cellular output. The function of the different classes of inhibitory neurons (INs) projecting to these subcellular compartments has been the subject of intense investigation^10–12^, yet how the structural plasticity of ISs supports these functions is largely unknown. Likewise, different classes of INs projecting to different subcellular compartments have been implicated in different aspects of hippocampal learning^13–21^, but if and how structural IS plasticity in these compartments relate to learning has not been established.

Longitudinal two-photon (2 P) optical imaging enabled investigating long-term dynamics of excitatory synaptic structural plasticity in the brain of live animals and led to a leap in understanding of brain plasticity and its role in learning and recall^22–24^. By comparison, the structural plasticity of ISs is understudied. Moreover, the vast majority of previous work mostly focused on neocortical areas^25–30^, thus leaving other brain areas key for learning and memory—such as the hippocampus—unexplored.

Here, we confronted this knowledge gap by labeling ISs impinging on PNs located in the dorsal hippocampal CA1 area (dCA1) of mice and by tracking them over two weeks by using deep-brain 2 P longitudinal optical imaging. This allowed us to simultaneously monitor ISs located on basal dendritic arborizations, somata, and AIS of dCA1 PNs and to characterize the long-term structural dynamics of these ISs. Then, we compared dynamics of inhibitory and excitatory synapses—by using dendritic spines as a proxy—during baseline conditions and upon learning. Size and temporal dynamics of ISs showed a very strong compartmentalization with soma ISs being the largest and most dynamic ISs. Newborn dendritic ISs were more dynamic than newborn dendritic spines. Trace fear conditioning led to compartmentalized changes in IS’s long-term temporal dynamics. Finally, we used mathematical modeling to investigate the circuit mechanisms underlying stabilization of AIS ISs upon learning of a hippocampal-dependent task.

## Results

### Longitudinal tracking of ISs in different subcellular compartments of dCA1 PNs

**Fig. 1:**
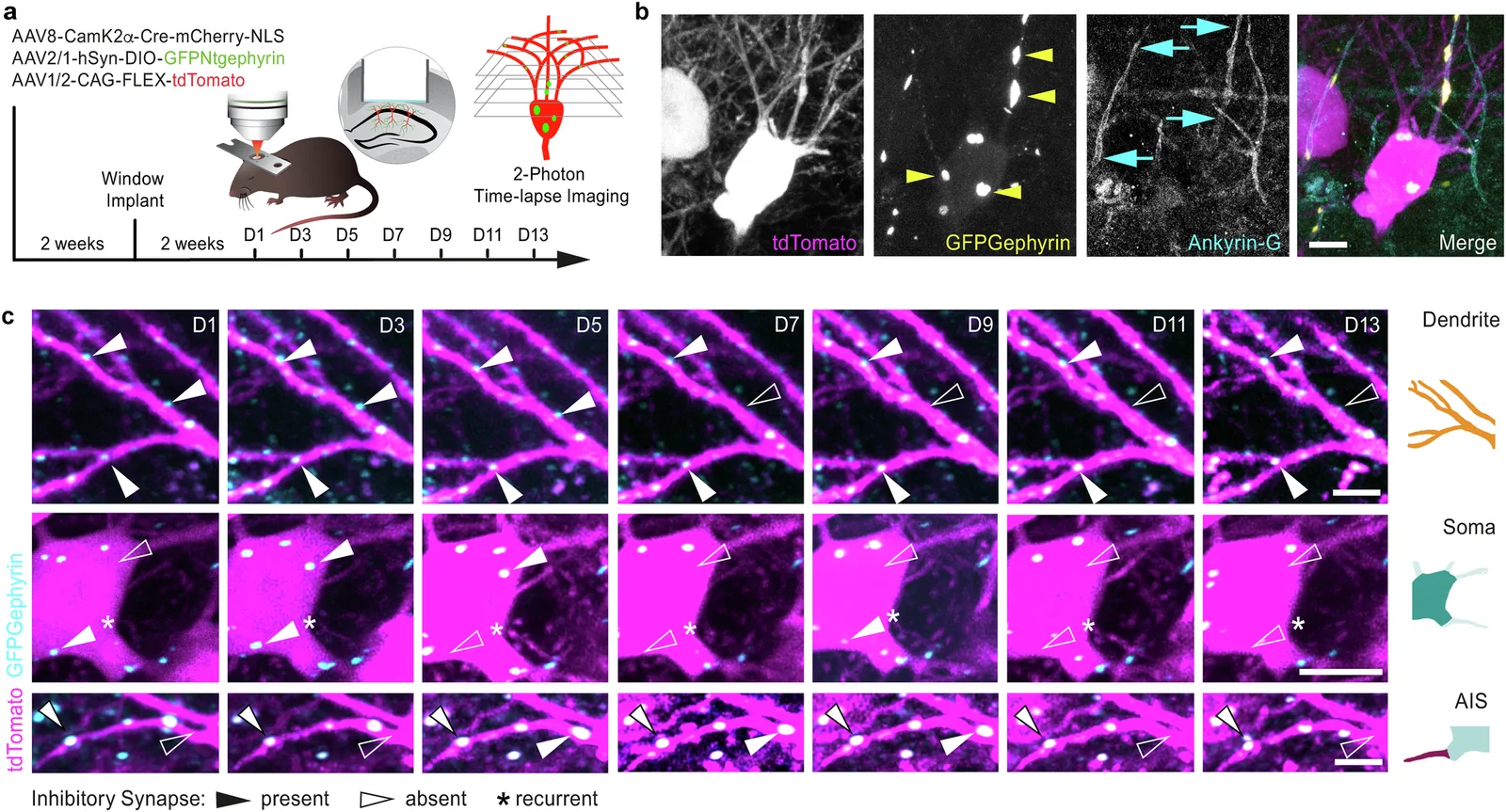
Longitudinal tracking of dCA1 PNs’ ISs in different subcellular compartments. **a** Schematic description of the strategy to label a sparse subset of dCA1 PNs and the ISs impinging on them as well as of the experimental timeline. Illustration adapted from^110^, CC BY 4.0.** b** Confocal images (Maximum Intensity Projection, MIP, of 55 slices, z-step 0.25 µm) of dCA1 PNs expressing GFP-Gephyrin and tdTomato and immuno-stained for ANK-G. Green triangles indicate ISs, blue arrows indicate a neurite positive for ANK-G. Scale bar, 5 μm. **c** Two-photon time series of dCA1 PN subcellular compartments expressing GFP-Gephyrin and tomato over 13 days. Solid and empty triangles indicate present and absent synapses, respectively. Asterisks mark recurrent synapses. (MIPs of 15–40 slices, z-step 1 µm). Scale bars, 5 µm.

We adapted a method to genetically tag ISs in the brain of live mice^25,27^ and labeled PNs and the ISs impinging on them. We transduced the dCA1 of 6 C57Bl6 mice with three Adeno Associated Viruses encoding for (i) a Cre-recombinase under the control of the pan-excitatory neuronal promoter CaMKII alpha, (ii) a Cre-dependent, cell-filling, red fluorescent protein (tdTomato), and (iii) a Cre-dependent, green fluorescent protein (GFP) fused to the GABAergic postsynaptic protein Gephyrin (Fig. 1a). We thus detected ISs on basal dendrites, somata, and AIS of a subset of dCA1 PNs. AIS ISs were located on the axon initial segment—identified as the thinner arborization originating from the soma—and distinguished from dendritic ISs by their larger size ( ~ 1 mm^2^ vs. 0.3 mm^2^)—consistent to axo-axonic synapses^31–35^. AIS ISs as identified in vivo, overlapped with Ankyrin G immunostaining (Fig. 1b and Fig. S1a), whereby >90% of Gephyrin-GFP+ puncta were also positive for vesicular GABA Transporter (vGAT) immunofluorescence (Fig. S1b, c). vGAT colocalization with Gephyrin-GFP exceeded chance levels in both somatic and dendritic layers (Fig. S1d, e). We used longitudinal 2 P optical imaging to image ISs impinging on these subcellular compartments. (Fig. 1c) and tracked basal dendritic, somatic, and AIS ISs (Fig. S1f) for a duration of 13 days. In total, we sampled 476 dendritic, 472 somatic, and 169 AIS independent inhibitory postsynaptic sites, i.e. the positions on a PN where we detected an IS at least once during the experiment (Fig. S1g).

### IS’s area is compartmentalized on dCA1 PNs

**Fig. 2:**
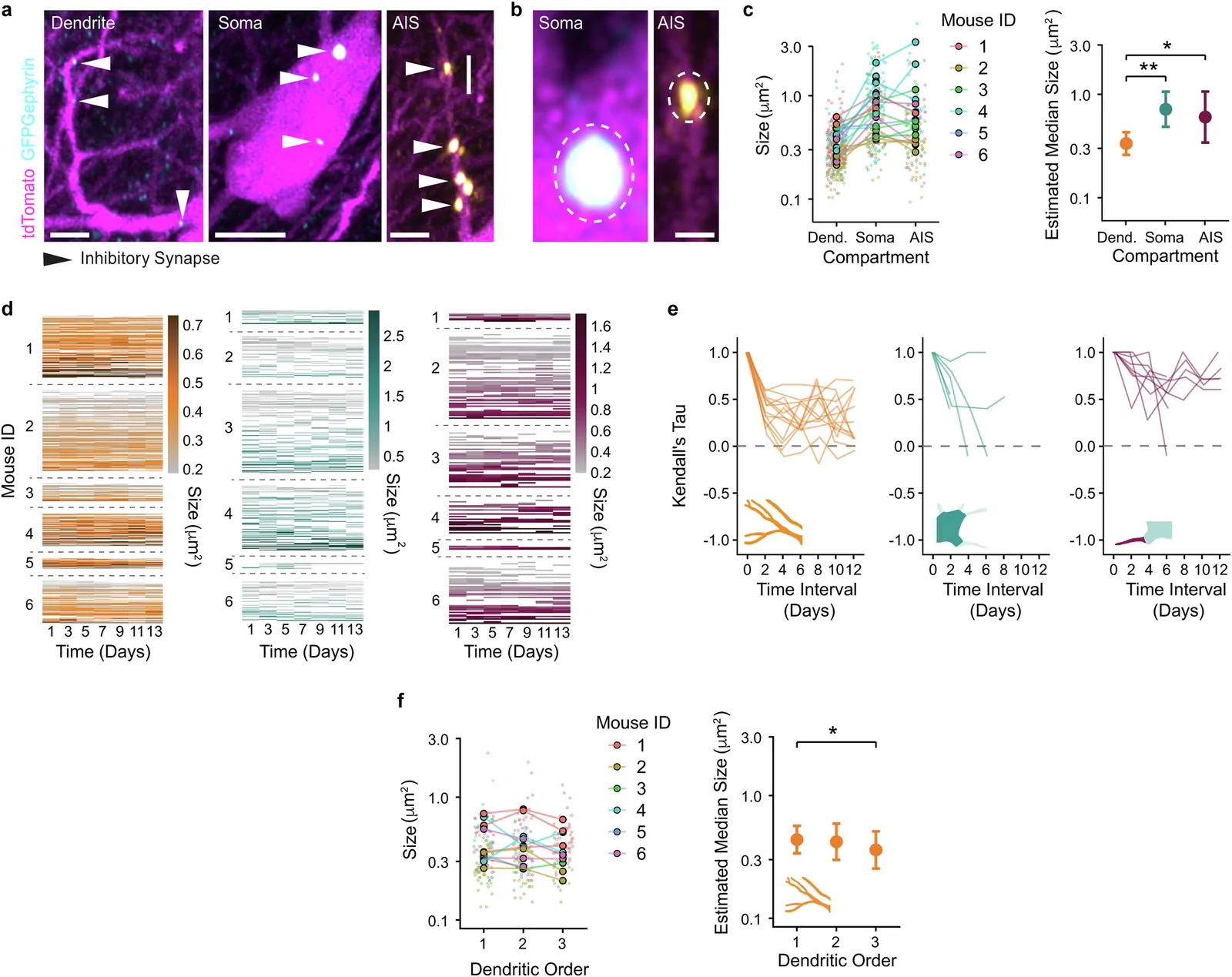
Compartmentalized properties of inhibitory synapse size. **a** Two-photon maximum-intensity projections (MIPs; 15–40 slices, z-step 1 µm) of GFP-Gephyrin-positive inhibitory synapses (ISs; yellow on dorsal CA1 pyramidal neurons (PNs; dTomato, magenta). Triangles indicate synapses. Scale bars, 5 µm. **b** Single z-planes of GFP-Gephyrin-positive ISs on PNs. Ovals indicate regions of interest (ROIs). Scale bars, 2 µm. **c** Day 1 IS areas (log scale) across basal dendritic, somatic, and AIS compartments. Left: ISs (dots), per-neuron means (circles), mice (colors); *n* = 363 basal dendritic ISs/18 neurons, 163 somatic ISs/27 neurons, and 112 AIS ISs/17 neurons from 6 mice (biological replicates). Right: model-estimated medians (points), 95% confidence intervals (CIs; error bars), from a linear mixed-effects model. Two-sided CR2 contrasts used small-sample-adjusted df and closed-testing adjustment for multiple comparisons: Basal dendritic (orange) vs. somatic (teal), t(4.00) = − 6.79, adjusted *p* = 0.0024**, median ratio = 0.48 [95% CI, 0.35–0.65]; basal dendritic vs. AIS (purple), t(4.23) = − 3.13, adjusted *p* = 0.033*, median ratio = 0.56 [0.34–0.93]; somatic vs. AIS, t(3.58) = 2.09, adjusted *p* = 0.113, median ratio = 1.18 [0.94–1.49]. **d** IS areas over 13 days (basal dendritic, orange; somatic, teal; AIS, purple). Bars represent ISs; color intensity reflects size. Measurements: 2494 basal dendritic, 1047 somatic, 743 AIS. **e** IS-size stability (Kendall’s τ between Day 1 and later intervals). Each trajectory represents τ per neuron. Dashed line, τ = 0. Only τ values with ≥5 contributing compartment-specific ISs are shown. **f** Day 1 basal dendritic IS sizes (log scale) across dendritic orders (DOs) 1–3. Model-estimated medians (points), 95% CIs (error bars), from a linear mixed-effects model. *n* = 117 ISs/16 neurons/6 mice for DO1; 106/12/5 for DO2; 83/10/5 for DO3 (mice =biological replicates). Two-sided CR2 contrasts used small-sample-adjusted df and closed-testing adjustment for multiple comparisons. DO1 vs. DO2, t(3.54) = 0.59, adjusted *p* = 0.593, ratio = 1.04 [95% CI, 0.85–1.27]; DO1 vs. DO3, t(3.46) = 5.66, adjusted *p* = 0.0499*, ratio = 1.21 [1.10–1.34]; DO2 vs. DO3, t(3.21) = 2.46, adjusted *p* = 0.086, ratio = 1.17 [0.96–1.41]. Mixed-effects models accounted for the nested data structure described in Methods. Source data are provided as a Source Data file.

To quantify the area of ISs in different subcellular compartments of dCA1 PNs (Fig. 2a), we identified circular regions of interest centered on Gephyrin-GFP-positive puncta and counted the number of fluorescent pixels after thresholding using Otsu’s method (Fig. 2b). We made 2494 measurements of basal dendritic, 1047 measurements of somatic, and 743 measurements of AIS ISs across the 13 day-imaging period. We treated all Gephyrin-GFP positive puncta as single ISs, while larger puncta likely contain clusters of smaller underlying ISs too close to each other to be resolved by in vivo 2 P microscopy. The IS area distributions were non-Gaussian and skewed towards higher values (Fig. S2a). At Day 1, basal dendritic ISs were significantly smaller than somatic and AIS ISs (Fig. 2c), and the same relative size pattern was observed across the imaging period (Fig. S2b).

As IS area correlates with the number of synaptic receptors and to synaptic transmission efficacy^36–38^, tracking the areas of hundreds of ISs through time (Fig. 2d) enabled us to investigate the temporal stability of the patterns of synaptic inhibition on PNs. To assess the temporal consistency of IS’ sizes over time, we quantified pairwise rank stability using Kendall’s τ for IS areas measured both at the first and subsequent time points within individual neurons. For dendritic IS and AIS, Kendall’s τ remained consistently positive and moderately high across 13 days, indicating a moderate preservation of IS size patterns over time (Fig. 2e, left and right). This measurement was much less reliable in somatic IS, due to their lower stability (Fig. 2e, middle). On Day 1 the size of basal dendritic IS was smaller in the third than in the first dendritic order (DO) (Fig. 2f), and the same relative size pattern was observed across the imaging period (Fig. S2c).

### Long-term dynamics of ISs are compartmentalized on dCA1 PNs

**Fig. 3:**
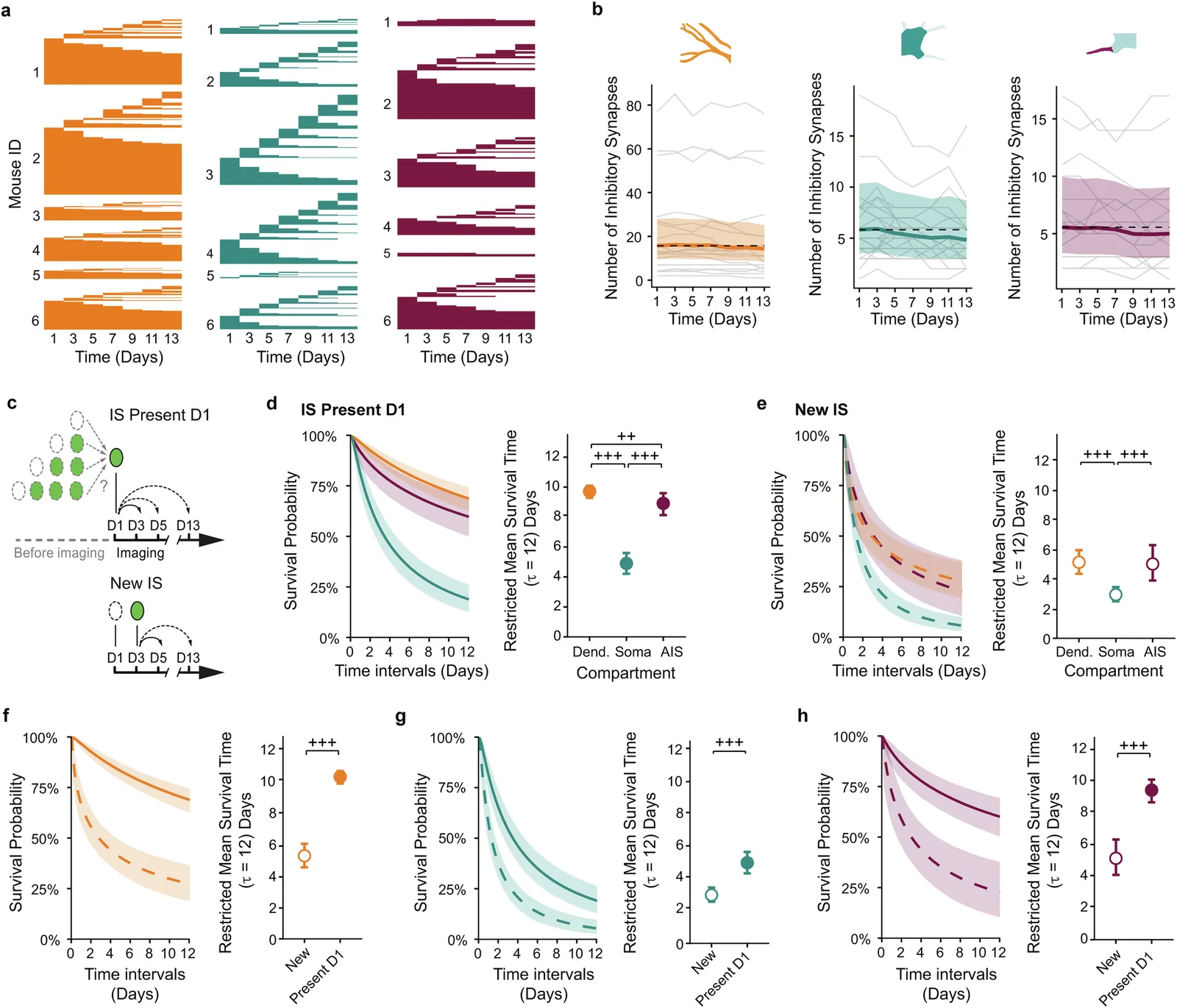
Compartmentalized properties of inhibitory synapse dynamics. **a** ISs across imaging days, ranked by survival. Bars represent ISs; colors denote basal dendritic (orange), somatic (teal), AIS (purple) compartments.** b** IS counts per neuron across days. Thin lines, neurons; thick lines, posterior medians from a Bayesian negative-binomial mixed-effects model; ribbons, 95% credible intervals (CrIs); dashed lines, Day 1 posterior medians. *n* = 18 basal dendritic, 27 somatic, and 17 AIS neurons (16 at Day 7) from 6 mice (biological replicates).** c** Schematic distinction between ISs present at Day 1 and formed thereafter.** d**,** e** Survival and restricted mean survival time (RMST; τ = 12 days) for ISs present at Day 1 (d, solid) or formed thereafter (e, dashed), estimated using Bayesian lognormal accelerated failure-time mixed-effects models. Lines/points show posterior medians; ribbons/error bars, 95% CrIs. **d** *n* = 363 basal dendritic ISs/18 neurons, 163 somatic ISs/27 neurons, and 112 AIS ISs/17 neurons, all from 6 mice (biological replicates). Posterior RMST differences (Δ), probability of direction (pd): basal dendritic - somatic, 4.78 days, pd > 0.999^+++^; somatic–AIS, −3.82 days, pd > 0.999^+++^; basal dendritic–AIS, 0.96 days, pd = 0.985^++^. **e** *n* = 169 basal dendritic ISs/15 neurons and 347 somatic ISs/27 neurons, each from 6 mice, and 72 AIS ISs/16 neurons from 5 mice. RMST differences: basal dendritic-somatic, 2.46 days, pd > 0.999^+++^; somatic–AIS, −2.33 days, pd > 0.999^+++^; basal dendritic–AIS, 0.13 days, pd = 0.566. Day 1 versus newborn ISs in basal dendrites (**f**; 363 ISs/18 neurons vs. 169/15, 6 mice), soma (**g**; 163/27 vs. 347/27, 6 mice), and AIS (**h**; 112/17 from 6 mice vs. 72/16 from 5 mice). RMSTs used the same models as (**d**, **e**); lines/points show posterior medians and ribbons/error bars 95% CrIs. RMST differences (Day 1–newborn): basal dendritic, 4.77 days; somatic, 2.45 days; AIS, 3.92 days (all pd >0.999^+++^). Mice are biological replicates throughout. Source data are provided as a Source Data file.

While at every time point we observed IS gain and loss in all compartments (Fig. 3a), we did not detect a significant drift in overall IS numbers *per* neuron across 13 days (Fig. 3b**;** Fig. S2d). Somatic ISs were significantly less stable than dendritic or AIS ISs, with a remarkably faster decay and a strongly reduced survival. This compartment-wise difference was significant both for ISs present at the first day of imaging (pre-existing)—representing a heterogeneous population spanning a broad range of ages (Fig. 3c, d)—and for newborn ISs—constituting a more homogeneous population restricted to lifetimes shorter than 48 h (Fig. 3c, e). Newborn dendritic spines are less stable than older ones, because they tend to miss functional synapses^39–44^. Similarly, newborn ISs disappeared faster than pre-existing ISs in all three subcellular PNs’ compartments (Fig. 3f–h), indicating that ISs are also more susceptible to be eliminated early during their maturation.

As high turnover of somatic ISs could result from synapses persisting but shifting their position, we aimed to measure the motion of persistent ISs, to test whether this motion could be confused for turnover. We thus sampled 120 ISs as well as stable landmarks as often as our technique allowed (every 10 min) in a new set of mice (Fig. S3a–c) and retrieved their spatial coordinates (Fig. S3d). We then projected these coordinates in the same three-dimensional frame (Fig. S3e) to measure the distances of each IS and landmark at each time point to itself at any other time point. There were no differences between the distances of ISs and landmarks in all subcellular compartments (Fig. S3f), showing that the position of persistent ISs is equally stable in all compartments. Thus, the higher turnover of somatic ISs compared to other subcellular compartments cannot originate from higher positional fluctuations.

### Longitudinal tracking of dendritic spines as proxies for excitatory synapses

**Fig. 4:**
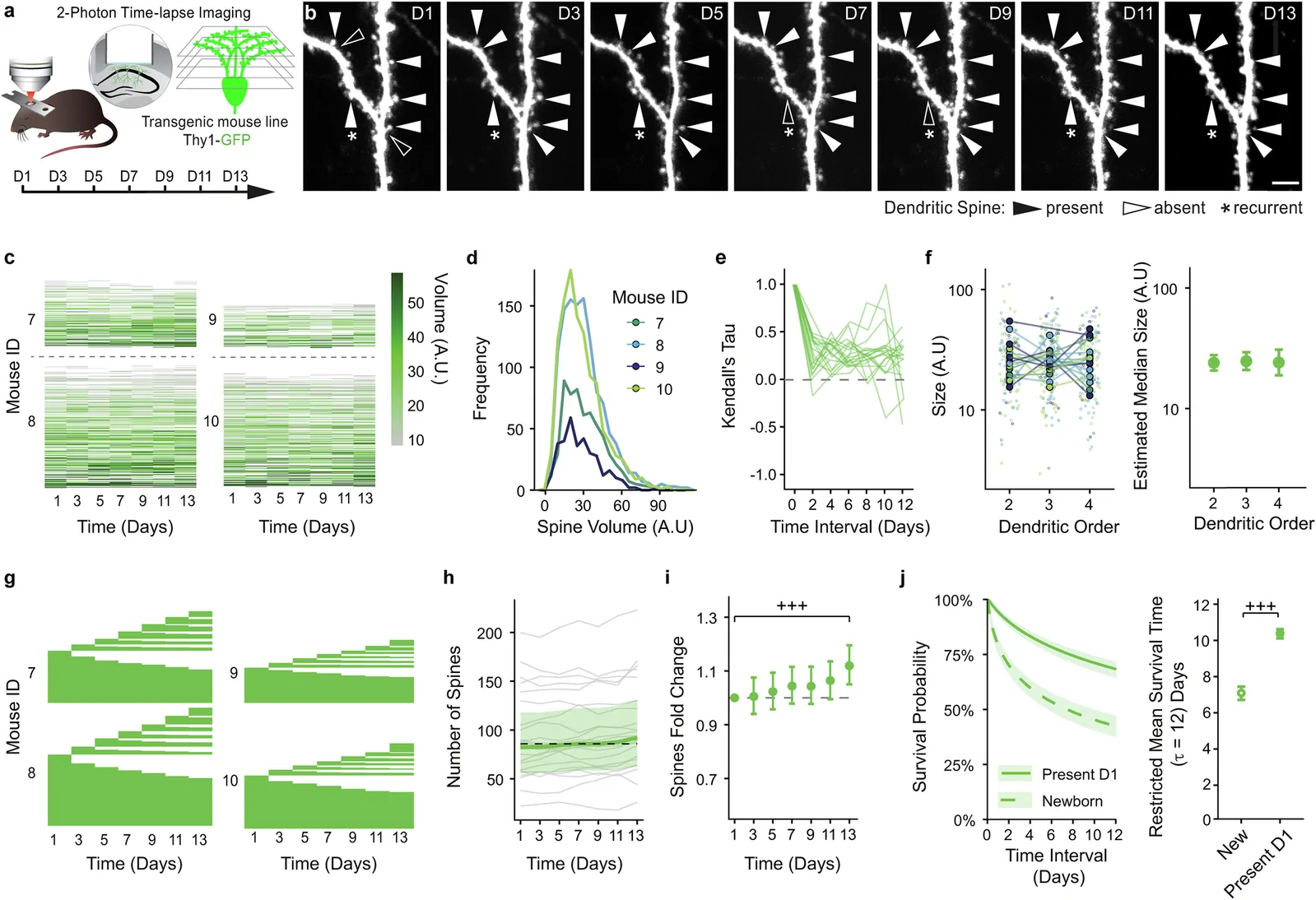
Longitudinal tracking of dCA1 PNs dendritic spines as proxies for excitatory synapses. **a** Schematic of sparse dCA1 pyramidal neuron (PN) labeling by somatic GFP expression in Thy1-GFP mice and experimental timeline. Illustration adapted from^110^, CC BY 4.0. **b** Two-photon time series of GFP+ dCA1 PN basal dendrites/spines. Solid/open triangles, present/absent spines; asterisks, recurrent spines. MIPs, 10 slices, z-step 1 µm. Scale bar, 5 µm. **c** Spine volumes across 13 days. Bars represent individual spine trajectories; color intensity reflects volume. 871 measured spine volume trajectories from 21 neurons in 4 mice. **d** Spine-volume distributions per mouse. **e** Spine-size stability (Kendall’s τ, Day 1 versus later days). Trajectories represent neurons (*n* = 20); dashed line, τ = 0. Only values with ≥5 paired spine measurements are shown. **f** Day 1 spine volumes across DOs 2–4. Model-estimated medians (points) and 95% CIs (error bars) from linear mixed-effects model *n* = 109 spines/16 neurons for DO2, 174/19 for DO3, and 124/17 for DO4, from 4 mice. **g** Spines ranked by birth date and survival; bars, individual spine trajectories. *n* = 3418 trajectories from 22 neurons in 4 mice. **h** Spine counts per neuron. Thick line, posterior medians (Bayesian negative-binomial mixed-effects model); ribbon, 95% CrI; dashed line, Day 1 median. *n* = 22 neurons from 4 mice.** i** Fold change in spine count per neuron relative to Day 1 from the model in h. Points, posterior median fold change; error bars, 95% CrIs. *n* = 22 neurons from 4 mice. Fold change at Day 13 = 1.12, pd = 0.999^+++^.** j** Survival and RMST (τ = 12 days) for spines present at Day 1 (solid) or formed thereafter (dashed), estimated using a Bayesian lognormal accelerated failure-time mixed-effects model. Lines/points, posterior medians; ribbons/error bars, 95% CrIs. *n* = 2044 Day 1 spines and 1374 newborn spines from 22 neurons in 4 mice. Δ = 2.74 days, pd >0.999^+++^. Mice are biological replicates throughout. Mixed-effects models accounted for the nested data structure described in “Methods”. Source data are provided as a Source Data file.

Previous work in the visual cortex demonstrated significant differences between excitatory and IS dynamics^25,26^, but still very little is known about these differences in the hippocampus. To compare the dynamics of dCA1 inhibitory and excitatory synapses, we tracked dendritic spines - as proxies for excitatory synapses—in basal dendrites of dCA1 PNs of Thy-GFP mice (Fig. 4a, b) for a duration of 13 days. The distribution of dendritic spines’ volumes (Fig. 4c) was non-Gaussian and skewed towards higher values (Fig. 4d) and the ordinal ranking of spine volumes per neuron showed a moderate consistency through time (Fig. 4e)—similar to dendritic ISs –. Spine volumes did not change significantly between DOs 2–4 (Fig. 4f**;** Fig. S4a).

When observing overall spine counts *per* neuron across 13 days (Fig. 4g), we found a significant increase on Day 13 in comparison to Day 1 (Fig. 4h, i). Newborn spines were on average less stable than pre-existing ones (Fig. 4j), consistent with previous work^44^ and similar to ISs.

### Stability of dendritic ISs and spines

**Fig. 5:**
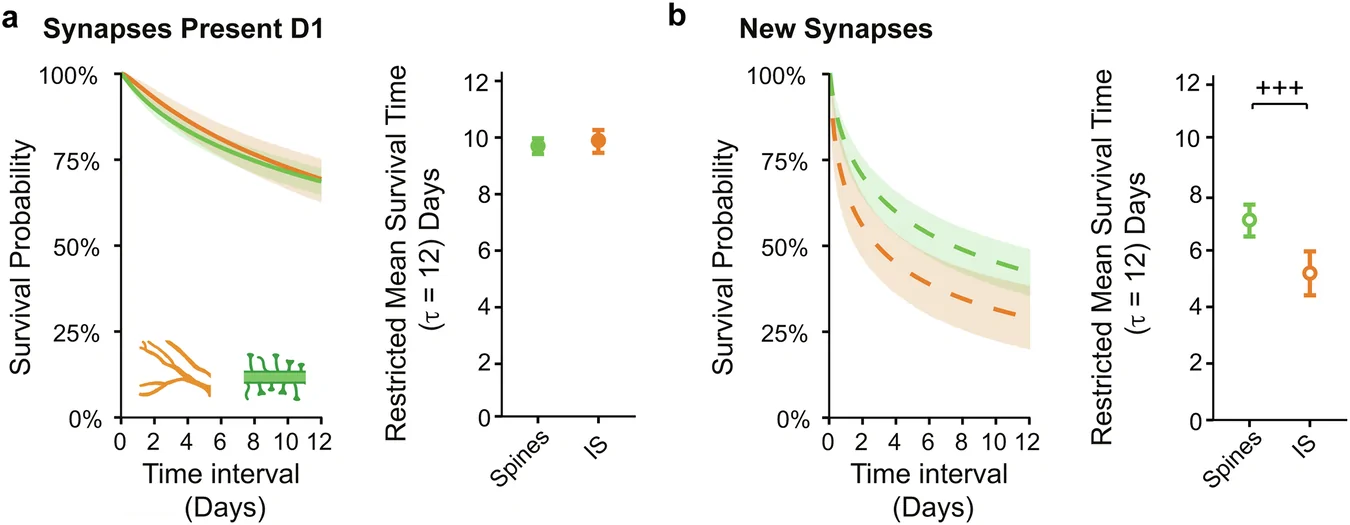
Newborn dendritic inhibitory synapses are less stable than newborn dendritic spines. **a** Survival and RMST (τ = 12 days) of Day 1 basal dendritic spines (green) and basal dendritic ISs (orange). Posterior medians (lines/points) and 95% CrIs (ribbons/error bars) from Bayesian lognormal AFT mixed-effects models. *n* = 2044 spines/22 neurons from 4 mice and 363 ISs/18 neurons from 6 mice. Spine–IS: Δ = −0.25 days, pd = 0.799.** b** Survival and RMST of newborn basal dendritic spines and basal dendritic ISs, calculated and displayed as in (**a**). *n* = 1374 spines/22 neurons from 4 mice and 169 ISs/15 neurons from 6 mice. Spine–IS: Δ = 1.68 days, pd = 0.995^+++^. Mice are biological replicates throughout. Mixed-effects models accounted for the nested data structure described in “Methods”. Source data are provided as a Source Data file.

When we compared the stability of ISs and spines in basal dendrites of dCA1 PNs, we found that synapses detected on the first imaging day were statistically indistinguishable in terms of survival (Fig. 5a). However, newborn dendritic ISs were significantly less stable than newborn spines (Fig. 5b).

**Fig. 6:**
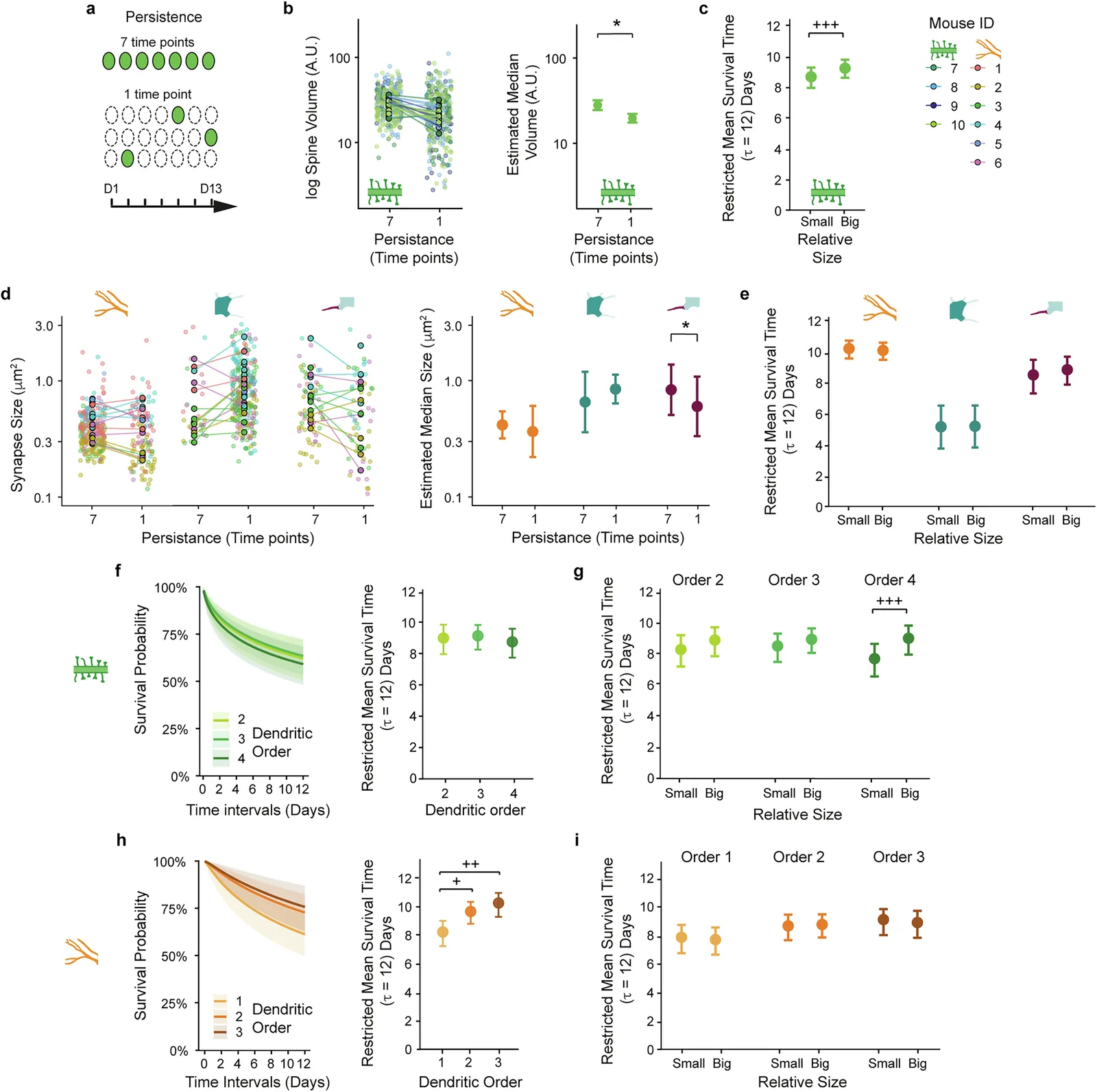
Differential relationship between size and stability between dendritic spines and inhibitory synapses. **a** Long- versus short-lived synapses. Day 1 sizes of long- and short-lived spines (**b**) and ISs (**d**). Left: log-scaled sizes; dots, synapses; circles, neuron means; colors, mice. Right: linear mixed-model medians (points) and 95% CIs (error bars). Two-sided CR2 contrasts used small-sample-adjusted df. **b** *n* = 291 long-lived spines/20 neurons/39 dendrites and 314 short-lived spines/21 neurons/41 dendrites from 4 mice; t(2.54) = 5.16, *p* = 0.021, ratio =  1.42 [95% CI, 1.12–1.82]. **d** Long-/short-lived samples: basal dendritic (orange), 253/115 ISs, 18/14 neurons, 6/6 mice; somatic (teal), 31/298 ISs, 15/27 neurons, 5/6 mice; AIS (purple), 67/54 ISs, 16/14 neurons, 6/4 mice. Contrasts: basal dendritic, t(3.94) = 1.03, *p* = 0.363, ratio = 1.10 [0.84–1.44]; somatic, t(3.55) = − 0.63, *p* = 0.567, ratio = 0.87 [0.46–1.66]; AIS, t(3.00) = 4.03, *p* = 0.027, ratio = 1.32 [1.06–1.64].** c**, **e–i** RMST (τ = 12 days) was estimated using Bayesian lognormal AFT mixed-effects models. **c**, **e** Compare the 25th (small)/ 75th (big) baseline-size percentiles: spines, 16.75/38.06 a.u.; basal dendritic ISs, 0.27/0.47 µm²; somatic ISs, 0.42/1.29 µm²; AIS ISs, 0.31/0.84 µm². **f–i** models additionally included a baseline size × dendritic-order (DO) interaction. Panels **f**, **h** show DO-specific survival and RMST at median size (spines, 26.07 a.u.; dendritic ISs, 0.36 µm²); **g**, **i** show within-DO RMST at the corresponding small/big sizes. Lines/points indicate posterior medians; ribbons/error bars, 95% CrIs. **c** *n* = 468 spines/21 neurons/42 dendrites/4 mice; Δ = 0.67 days, pd = 0.993⁺⁺⁺. **e** *n* = 363/163/112 dendritic/somatic/AIS ISs from 18/27/17 neurons and 6 mice. **f** DO2/3/4: *n* = 109/174/124 spines from 16/19/17 neurons, 26/34/22 dendrites and 4 mice. **g** Same n as in (**f**); Δ = 1.57 days, pd = 0.997⁺⁺⁺ at DO4. **h** DO1/2/3: *n* = 117/106/83 basal dendritic ISs from 16/12/10 neurons, 37/27/13 dendrites and 6/5/5 mice. DO2–DO1, Δ = 0.93 days, pd = 0.951⁺; DO3–DO1, Δ = 1.26 days, pd = 0.979⁺⁺. **i** Samples as in (**h**); no within-DO comparison had pd > 0.95. Mixed-effects models accounted for nested data structure as described in Methods. Mice were independent biological replicates throughout. Source data are provided as a Source Data file.

Long-lived dendritic spines (observed for 7 consecutive time points, Fig. 6a) were significantly bigger than short-lived ones (Fig. 6b) and, consistently, bigger spines survived significantly longer than smaller spines (Fig. 6c). For ISs, size and stability were differentially linked across compartments. Long-lived AIS ISs were significantly larger than short-lived ones, whereas no such size difference was detected for dendritic or somatic ISs (Fig. 6d). Finally, no size–survival relationship of AIS, dendritic, or somatic IS reached the significance threshold (Fig. 6e).

We then investigated whether synaptic size and/or DO of pre-existing synapses predicted their survival. For dendritic spines, population-level predictions from a hierarchical survival model with an interaction between size and DO showed no significant effect of DO on spine survival (Fig. 6f). Within individual DOs, larger spines survived longer than smaller ones only in order 4 (Fig. 6g). Interestingly, survival of dendritic ISs differed systematically across DOs, with DO 1 being less stable than DO 2 and 3 ISs (Fig. 6h). However, we did not find any significant survival differences between small and big dendritic ISs across DOs (Fig. 6i). This suggests DO, rather than size, as the stronger determinant of dendritic ISs stability.

### Learning changes IS dynamics in dCA1

**Fig. 7:**
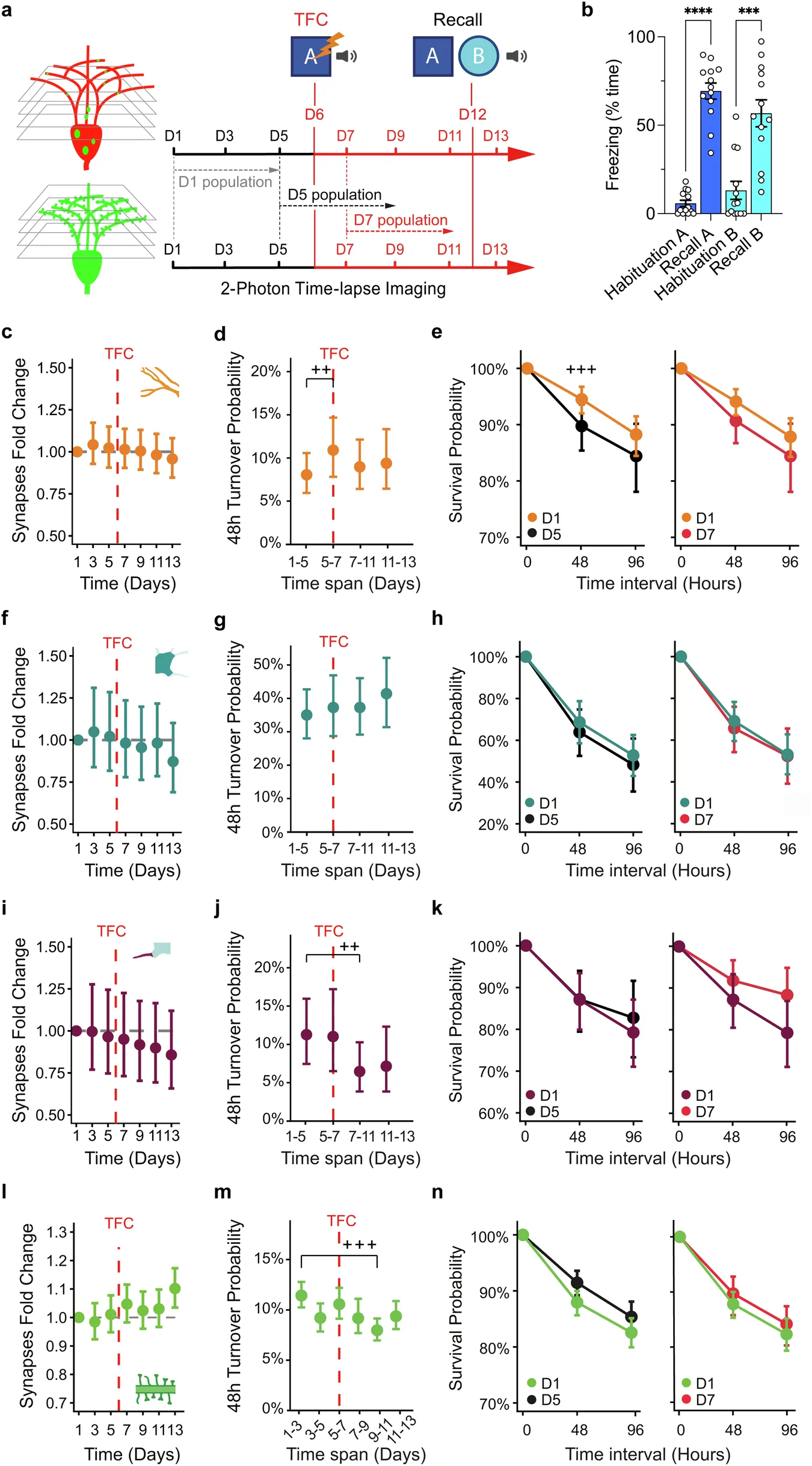
Learning a fear association changes dCA1 synaptic dynamics. **a** Schematic of IS and basal dendritic-spine labeling and experimental timeline.** b** Freezing during habituation and recall in Contexts A and B. Circles, mice; bars, means; error bars, s.e.m.; *n* = 13 mice. Friedman test followed by two-sided Dunn’s multiple-comparisons tests, habituation versus recall: Context A, rank-sum difference = −28.50, Z = 4.329, adjusted *p* < 0.0001; Context B, rank-sum difference = −23.50, Z = 3.570, adjusted *p* = 0.0007.**c–n**, Points and error bars show posterior medians and 95% CrIs from Bayesian mixed-effects models with TFC and control mice (groups). pd measures change within TFC; interaction pd tests whether change differed from controls. Red dashed line, time of TFC. TFC vs. control, n (mice/neurons): **c–e**, basal dendritic ISs, 8/28 vs. 6/18; **f–h** somatic ISs, 8/28 vs. 6/27; **i–k** AIS ISs, 7/19 vs. 6/16; **l–n** basal dendritic spines, 5/26 vs. 4/22. **c**,** f**,** i**,** l** Day 1-normalized synapse numbers per neuron. Negative-binomial models tested day × group. Spine number increased from Day 1 to Day 13 within TFC mice (fold change = 1.10 [1.04–1.17], pd = 0.999), but this increase did not differ from controls (interaction pd = 0.629).** d**,** g**,** j**,** m** Binomial models estimated 48-h turnover over Day intervals. Basal dendritic-IS turnover increased during Days 5–7 compared with Days 1–5 (Δ = 0.028 [0.004–0.057], pd = 0.989⁺⁺; interaction pd = 0.976). AIS turnover decreased during Days 7–11 compared with Days 1–5 (Δ = −0.047 [ − 0.089 to −0.010], pd = 0.993⁺⁺⁺; interaction pd = 0.955). Basal dendritic-spine turnover decreased from Days 1–3 to Days 9–11 (Δ = −0.034 [ − 0.047 to −0.023], pd > 0.999⁺⁺⁺; interaction pd = 0.994).** e**,** h**,** k**,** n** Binomial models estimated 48- and 96-h survival for synapses present on Day 1 versus Day 5 (left) and Day 1 versus Day 7 (right). At 48 h, basal dendritic-IS survival was lower for synapses present on Day 5 than Day 1 (Δ = −0.047 [ − 0.087 to −0.015], pd = 0.997⁺⁺⁺; interaction pd = 0.964). Mice were biological replicates throughout; a detailed mixed-effects model structure is described in Methods. Source data are provided as a Source Data file.

To investigate how learning affects structural synaptic plasticity in the hippocampus, we tracked basal dendritic spines as well as basal dendritic, somatic, and AIS ISs in mice subjected to Trace Fear Conditioning (TFC) (Fig. 7a). TFC is a strong one-trial hippocampal-dependent learning task, and mice significantly froze above baseline levels during context and tone recall (Fig. 7b).

To test whether TFC led to a change in the number of synapses we compared the number of synapses *per* neuron using a negative binomial mixed effects model to test changes induced by TFC towards baseline and control. Importantly, TFC effects were marked as significant when they showed a significant change upon TFC compared to baseline and when this change was significantly different to the change between the same epochs in the control condition. We did not find any significant changes in ISs numbers across (Day 5–7) or after TFC (Day 7-13) (Fig. 7c, f, i; Fig. S5a–c). For spines we found an evident increase in number *per* neuron during Day 9–11. This increase was, however, not significantly different from the increase we found in the control condition (Fig. 4h, i) and thus could not be attributed to TFC (Fig. 7l and Fig. S5d).

To examine the effect of TFC on synaptic stability in the different subcellular compartments, we used binomial Bayesian models to compare population-level effects of 48 h turnover (Fig. S5e–h) as well as survival probabilities before across and after TFC. As we did not find significant drift in baseline (Day 1–5) or post-TFC (Day 7–11) for IS turnover (Fig. S5i-k), we averaged posterior draws across timepoints within baseline (Day 1–5) and post-TFC (Day 7–11) epochs to obtain more robust estimates (Fig. 7d, g, j). Our turnover models also included the control group to distinguish true TFC effects from baseline drift. Dendritic ISs showed a significant increase in turnover upon TFC (Fig. 7d). Dendritic ISs present prior to TFC displayed a significant decrease in survival upon TFC (Fig. 7e, left) and this destabilization trend was maintained in dendritic ISs present post TFC (Fig. 7e, right), in comparison to baseline. We did not detect any significant effect of TFC on somatic ISs (Fig. 7f–h; Fig. S5j). Interestingly, we detected a significant decrease in AIS IS turnover only after TFC (Fig. 7j). Consistently, survival of AIS ISs present before TFC was similar to baseline survival up to 24 h after TFC (Fig. 7k, left) while AIS ISs present the day after TFC tended to survive longer than baseline—albeit not reaching significance against control (Day 7, Fig. 7k, right)—. Finally, we did not apply averaging of posterior draws into different epochs for spines, since they showed baseline drift in turnover. While we found a significant decrease in spine turnover between the Day 1–3 (but not Day 3–5) and the Day 9–11 interval, the presence of baseline drift precludes confident attribution of post-TFC changes to learning. We did not find any significant differences of 48 h or 96 h loss for spines present at day 1 to spines present at Day 5 or Day 7 (Fig. 7n).

Overall, our data shows that learning a fear association significantly affects IS dynamics, leading to opposite outcomes for ISs located in different subcellular compartments: rearrangement of ISs located on the dendrites and stabilization of ISs located on the AIS of dCA1 PNs.

### Computational modeling to investigate the role of AIS-projecting INs on the local network inhibitory connectivity patterns

**Fig. 8:**
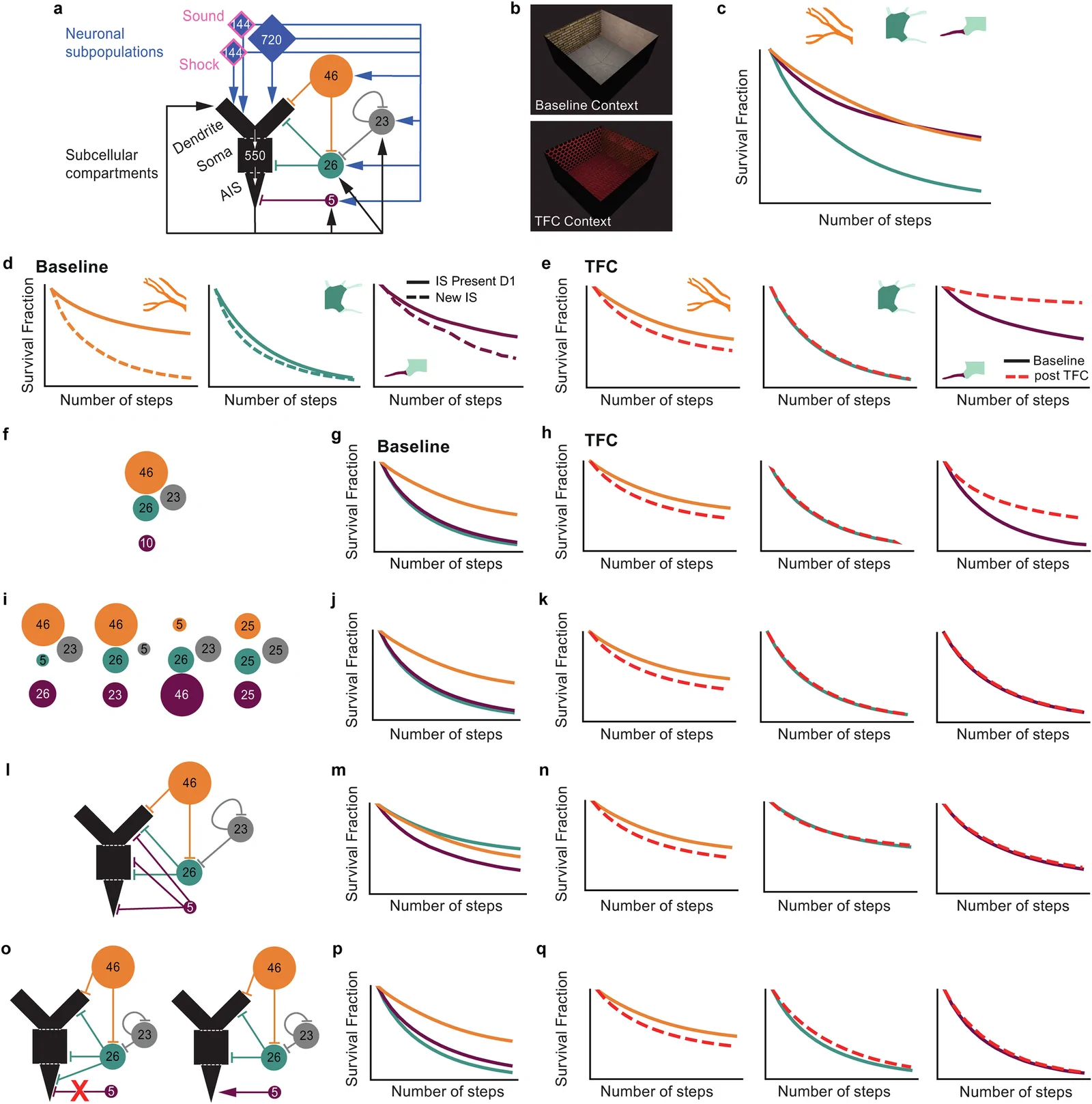
Computational modeling to investigate the role of AIS-projecting neurons on the local network inhibitory connectivity patterns. **a** Schematic description of the model of the local neuronal network. The model includes 1008 excitatory input PNs (blue)—of which 288 only active during TFC Context to indicate sound and shock (pink outline) -, (ii) 550 excitatory output PNs—with distinct dendrite, soma and AIS compartments – (black), and 4 types of INs classified according to their connectivity to subcellular compartments of output neurons (46 dendritic-projecting, orange; 26 mostly somatic-projecting, teal; 5 AIS-projecting, purple; 23 IN-projecting, gray).** b** Computer renderings of the environments the virtual agent navigated during baseline (top) or conditioning (bottom). **c** Survival Fractions of modeled dendritic (orange), somatic (teal), and AIS (purple) ISs during the baseline period. **d** Comparisons of the Survival Fractions of modeled dendritic (orange, left), somatic (teal, middle), and AIS (purple, right) pre-existing (solid lines) versus new ISs (dashed lines). **e** Comparisons of the Survival Fractions of modeled dendritic (orange, left), somatic (teal, middle), and AIS (purple, right) ISs during the baseline period versus after TFC (dashed red lines). Doubling of the number of AIS-projecting INs (**f**) leads to baseline destabilization of AIS ISs (**g**) but does not affect TFC-induced AIS ISs stabilization (**h**). Changing the relative proportion of AIS-projecting INs (**i**) leads to baseline destabilization of AIS ISs (**j**) and prevents TFC-induced AIS ISs stabilization (**k**). Allowing AIS-projecting INs to project to all compartments (**l**) leads to moderate baseline destabilization of AIS and strong stabilization of somatic ISs (**m**). It also prevents TFC-induced AIS IS’ stabilization (**n**). Removing AIS-projecting INs projections or making them excitatory (o) leads to baseline destabilization of AIS ISs (**p**) and prevents TFC-induced AIS IS stabilization (**q**).

AIS-projecting INs are ideally positioned to control the output of dCA1 PNs^33,45^, they have been shown to affect activity patterns of PNs in different brain areas and to lead to long-term plasticity changes^46–49^. We thus wanted to investigate the role of these neurons on structural IS plasticity. To this aim we generated a neural circuit model simulating the main features of the local dCA1 connectivity and focused on the changes in dynamics of structural IS plasticity occurring when we specifically changed parameters of AIS-projecting INs. Our model included (i) excitatory input PNs (Fig. 8a, blue) some only active during the training paradigm (Fig. 8a, pink outline), (ii) excitatory output PNs—with distinct dendrite, soma, and AIS compartments—(Fig. 8a, black), and four types of INs classified according to their connectivity to output neurons (Fig. 8a, orange for dendritic-projecting, teal for mostly somatic-projecting, purple for AIS-projecting, and gray for IN-projecting) and whose proportions were derived from literature^9,10,50–62^. Importantly, as we also wanted to test the effect of learning on IS plasticity, we used a reinforcement learning setup that mimicked our experimental TFC protocol (Fig. 8b). The simulated circuit model was used to control the behavior of an agent navigating a virtual environment. During baseline, the agent navigated the environment retrieving random rewards, then it was exposed to a novel environment where the stimulation of sound and shock neurons simulated the TFC protocol by delivering a punishment. We tuned the amplitudes of the stochastic contribution to rewiring to qualitatively match the turnover curves obtained experimentally during baseline (Fig. 8c, d).

Then we fixed the model parameters and applied the TFC protocol. The resulting turnover dynamics in the model were consistent with our experimental results, with a TFC-dependent destabilization of dendritic, no significant changes in somatic and a stabilization of AIS ISs (Fig. 8e, from left to right, respectively).

We then investigated the role of AIS-targeting INs on ISs structural dynamics by disrupting the circuit architecture as well as unique features of AIS-projecting INs and by quantifying the resulting changes in IS survival. First, we simply increased the number of AIS-projecting INs (Fig. 8f). While this led to a sharp drop in survival of AIS ISs during baseline (Fig. 8g), it did not change the effect of TFC (Fig. 8h). Second, we changed the relative proportions of AIS-projecting INs while keeping their total number constant (Fig. 8i). This also led to a sharp drop in survival of AIS ISs during baseline (Fig. 8j), and prevented their TFC-induced stabilization (Fig. 8k). Then we changed the connectivity of the AIS-projecting INs in different ways. First, we allowed these INs to project to all compartments (Fig. 8l). This led to de-stabilization of AIS and a marked stabilization of somatic ISs (Fig. 8m); moreover, this prevented TFC-induced stabilization of AIS ISs (Fig. 8n). Second we eliminated AIS ISs - and used soma-projecting INs to inhibit the AIS - or made the projections of AIS-projecting INs excitatory (Fig. 8o). This led to a sharp drop in baseline survival of AIS ISs (Fig. 8p), and also prevented TFC-induced stabilization (Fig. 8q).

## Discussion

### Longitudinal tracking of ISs in different subcellular compartments of dCA1 PNs

Investigating dynamics of excitatory synaptic structural plasticity in the brain of live animals lead to a leap in our understanding of brain plasticity and its role in learning and recall^22–24^. By comparison IS structural plasticity is understudied and previous studies tackling it in rodents mostly focused on neocortical areas^25–30^. Here, we labeled and tracked ISs and dendritic spines on dCA1 PNs over two weeks during baseline and upon learning a hippocampal-dependent memory task. Tracking the persistence of ISs through time and the stability of their size patterns enabled us to investigate the long-term efficacy of synaptic inhibition on PNs, as the efficacy of synaptic transmission on longer time scales is a function of the physical persistence and size of a synapse^36–38^. Finally, we used quantitative mathematical modeling to predict the properties of AIS-projecting INs that might underlie learning-induced changes in IS dynamics.

### Subcellular compartmentalization of IS size

The size of ISs is variable and we find some of this variability to map on specific neuronal subcellular compartments with basal dendritic being smaller than somatic and AIS ISs. This compartmentalization could be due to different classes on INs projecting selectively to the dendritic arbors and somata—for instance bigger soma synapses could originate from CCK basket cells that reportedly form large boutons—^10,59,63–65^ or to different clustering of several ISs on the soma or AIS^32–34^ and could reflect different strengths of synaptic transmission.

### Subcellular compartmentalization of IS dynamics

ISs located in different subcellular compartments also showed different levels of stability, with dendritic being the most and somatic being the least stable ISs. The basal dendritic compartment in dCA1 is predominantly targeted by Ivy cells^66,67^, which exhibit slow, synchronizing persistent firing. Structural stability of these dendric ISs implies that the same dendritic segments receive inhibition reliably over time, maintaining a spatially consistent inhibitory map across the basal dendritic tree. This stability could set consistent segment-specific depolarization thresholds, broadening the dynamic range for signal integration and contributing to the role of dendrite-projecting INs in controlling local dendritic nonlinear integration and plasticity processes^11,68–70^.

Somatic ISs survived significantly less than dendritic and AIS ones. While we cannot fully exclude the contribution of extra-synaptic Gephyrin-GFP aggregates, the high turnover of somatic ISs was not due to fluctuations in the position ISs on the soma and it cannot be explained by clustering of several synapses—as clustering of synapses leads to apparent stability^71^—besides also AIS ISs are clustered but they do not show higher turnover. The high somatic IS turnover was surprising as it is unclear how a high turnover level might support synchronization of the activity in large populations of PNs^12,45,62,64,72–74^. Interestingly, high temporal variability of somatic inhibition might support—if not underlie—constant changes in the patterns of cellular plasticity^75^ and activity^76–78^ which is a very prominent phenomenon in dCA1. Moreover, turnover of dendritic and somatic ISs might have fundamentally different consequences on neuronal connectivity. In fact, dendritic-projecting INs mostly have a single synaptic contact on a postsynaptic dendritic segment^8^—thus loss of a single dendritic IS is slated to have a large effect on the excitability of a dendritic segment—while somatic-projecting INs have multiple ISs on the soma of the same postsynaptic PN^68^—thus loss of a single IS will have a smaller effect on the connectivity of pre- and postsynaptic partners–. This redundancy of inhibitory somatic connectivity might thus render the synchronization of PNs robust in the face of high somatic IS turnover.

By comparison, the high apparent stability of AIS ISs might stem from IS clustering in the AIS. In fact—as we previously characterized for dendritic spines^71^ –, apparent synapses made up of underlying clusters of real synapses will appear more stable than the underlying real synapses themselves. Thus, the similarity of stability between dendritic and AIS ISs we observe has to be interpreted with caution, as underlying real AIS ISs are more likely less stable than observed ones.

### Newborn dendritic ISs are less stable than newborn spines

Newborn dendritic ISs had shorter survival than newborn dendritic spines. Previous work in the visual cortex found that ISs are more dynamic than spines when tracked over consecutive imaging sessions^25,26^. In contrast, in the hippocampus, we found no significant difference in survival between pre-existing ISs and spines (i.e., synapses present at Day 1 of our experiment). This suggests that despite newborn ISs being less persistent, hippocampal ISs present at any given timepoint (for example Day 1) achieve comparable persistence to spines, possibly through a higher ratio of long-term survivors. One further consideration is that survival of ISs and dendritic spines is calculated by tracking objects with different shapes, which might bias tracking through time. In the future, it will be interesting to track ISs and dendritic spines in the same neurons to investigate the local relationship between these two classes of synapses to higher level of detail.

### The relationship between size, dendritic order and persistency in dendritic spines and ISs

A relationship between size and stability has been shown for dendritic spines—in that bigger spines tend to survive longer than smaller spines and spines become smaller before disappearing on the time scale of hours^79–81^. In our dataset dendritic spines persisting for 13 days were larger than the ones observed during a single timepoint and larger spines showed higher survival times, thus confirming that in the dCA1 larger spines—that generally contain larger synapses—survive longer. In contrast, dendritic and somatic ISs showed no detectable relationship between size and persistence, at odds with a previous report linking dendritic ISs size to persistence in the visual cortex^27^. This discrepancy may reflect differences in statistical power, in how IS’ size was quantified (fluorescent pixel area *versus* brightness), or in detection thresholds between the optical systems used in the different experiments. AIS ISs were the exception: persistent AIS ISs were significantly larger than transient ones, consistent with larger AIS puncta representing aggregate synapses. However, baseline size did not significantly predict AIS IS survival, likely due to the small number of AIS observations.

Interestingly, for ISs, survival was shortest in the most proximal DOs. While size predicts survival for spines, dendritic IS survival was related to dendritic order and we found no clear evidence for an association with size. As different interneuron classes innervate distinct parts of the basal dendritic tree—with Ivy-cell inputs preferentially targeting more distal branches—the faster dynamics of proximal ISs could reflect differences in interneuron composition across dendritic orders^10,62,82^.

### Learning significantly affects IS’ dynamics in dCA1

Previous work showed IS dynamics in the visual cortex upon sensory deprivation and in the motor cortex upon motor learning^25–29^, we now demonstrate learning-related changes in ISs in the dorsal aspect of the hippocampus, a brain area important for episodic learning.

TFC increased turnover of basal dendritic ISs, leading mainly to the destabilization of ISs present before TFC, without significant changes in IS’ numbers. This is consistent with previous work in the motor cortex^28^, and indicates that restructuring of the pattern of inhibition in the basal dendrites of dCA1 PNs supports learning of a fear association. Changes in AIS IS were of opposite sign. AIS IS turnover significantly decreased after TFC, leading to increased persistence of the inhibitory connectivity pattern in the AIS. Since both IS numbers and the survival of ISs present before TFC did not decrease upon TFC, the lower turnover post-TFC indicates that TFC genuinely stabilizes AIS ISs rather than filtering unstable ISs. Inhibition at the AIS can very effectively prevent the generation of action potentials^49,83–85^, thus the TFC-dependent stabilization of AIS inhibitory connectivity pattern should lead to stabilization of the newly formed PNs output pattern. Overall, differential control of dendritic and AIS inhibition by structural plasticity has the potential to effectively regulate the long-term changes in activity patterns of large populations of PNs.

Not finding significant differences in the dynamics of somatic ISs in response to TFC was unexpected, as the number axonal boutons of parvalbumin-expressing INs—which mainly inhibit the soma and peri somatic regions of PNs—increase in the motor cortex upon training^28^. A possible explanation of this mismatch might lay in the different kinetics of learning used in the two studies. In fact, Chen and colleagues used a behavioral paradigm lasting several days and the increase in boutons they detected was gradual, over several days. This would suggest that single-shot learning such as TFC does not elicit significant structural somatic ISs plasticity.

Finally, while learning a fear association affected the dynamics of ISs, its impact on dendritic spines in dCA1 PNs is harder to ascertain in our dataset. Previous studies demonstrated plasticity and learning-induced changes in spines dynamics in the neocortex^44,81,86–96^, but no previous work addressed the effect of fear learning on spine dynamics in the hippocampus in vivo. Moreover, while changes in dendritic spines’ density after fear conditioning have been reported in the past in the hippocampal CA1^97,98^, these results were based on comparison of different groups of mice and the magnitude of these changes was small (0,2 to 0,5 spines/um increase in density in basal dendrites). In our data, the high variability in both control and baseline turnover of dendritic spines makes any statistical comparison to the TFC group difficult to interpret. In particular, both control and TFC datasets show a similar time-dependent increase in dendritic spines’ number, which prevents us from making a quantitative statement as the contribution of TFC to this phenomenon. The same applies to the TFC dataset where decrease in turnover after TFC is significant when compared to the first but not the second baseline time point.

Further experiments should clarify whether different learning paradigms, including ones based on positive feedback or with longer time scales, have different effects on structural hippocampal synaptic dynamics.

### Computational modeling suggests properties of AIS-projecting INs underlying the learning-induced changes in IS structural connectivity patterns

We established a neural circuit model simulating the main features of single PNs and of local dCA1 connectivity, and reproducing the main features of in vivo IS dynamics. We then used this model to systematically investigate how properties of AIS IS might affect IS structural dynamics in the main subcellular compartments of PNs upon learning.

All manipulations of AIS-projecting INs led to baseline destabilization of AIS ISs, and none of the manipulations led to changes in TFC-induced destabilization of dendritic ISs. Stabilization of AIS ISs upon TFC—a prominent feature of the in vivo dataset – was disrupted by all manipulations of AIS-projecting INs but it was robust to doubling the number of these same neurons. We thus identified three basic local circuit features supporting TFC-induced stabilization of AIS ISs. The first is compartmentalization of structural inhibition, as changing the proportions of INs—and thereby the specific distribution of ISs along the compartments—prevented TFC-induced stabilization of AIS ISs. The second is sparseness of connectivity, as stabilization upon TFC only occurred when AIS ISs originated from a single class of INs and when this class only projected to the AIS. In fact, when AIS-projecting INs also projected to the soma or INs projecting to other compartments also project to the AIS there was no IS stabilization upon TFC. Finally, AIS ISs stabilization upon TFC was dependent on the activity of AIS-projecting INs to be inhibitory, as flipping the sign of these neuron’s activity abolished AIS IS stabilization. Overall, our mathematical modeling suggests that the lower abundance together with the specific connectivity and inhibitory action support the key role of AIS-projecting INs during learning-induced stabilization of AIS IS patterns.

Theoretical and experimental work has shown how the presence of dendrites and structured connectivity can improve computational aspects of biological and artificial neuronal networks^99–102^. In the future, it will be important to test how patterned structural synaptic turnover on hippocampal dendrites might underlie the function of the hippocampus in learning and spatial navigation.

## Methods

### Animals

Adult female and male, 8–12 week old C57BL6/N mice and heterozygous Thy1-eGFP [Jackson Laboratories Tg(Thy1-EGFP)MJrs] mice were used for IS and dendritic spine imaging, respectively. Mice had ad libitum access to water and food and were group-housed with a maximum of 5 mice per cage. Mice were held on a 12/12 h light/dark cycle in temperature and humidity-controlled holding rooms. Experiments were conducted during the 12-h light period. Mice were randomly assigned to experimental or control groups before the start of each experiment and each cohort consisted of both control and experimental mice. The Control-IS group included 6 mice (3 males and 3 females), the Control-spine group included 4 mice (2 males and 2 females), the TFC-IS group included 8 mice (3 males and 5 females) and the TFC-spine group included 5 mice (3 males and 2 females). All animal procedures conformed to Max Planck Society and Leibniz Association guidelines and they were approved by the governments of Bavaria and Saxony-Anhalt in the license of animal experimentation (42502-2-1738 LIN).

### Surgical procedures

Stereotaxic injections in the dCA1 (anteroposterior (AP)  =  −2.30 mm, mediolateral (ML)  =  1.80 mm, dorsoventral (DV)  =   − 1.40 mm) were performed according to standard procedures 2 weeks prior hippocampal window implantation. To label inhibitory post synapses with GFP and PNs with tomato, we injected intracranially a mixture of three Adeno Associated Viruses: AAV8-CamK2α-Cre-mCherry-NLS (UNC Vector Core, 3.5 × 10^12^ vg/mL), AAV2/1-hSyn-DIO-GFPNtgephyrin (Vector Biolabs, 3.6 × 10^12^ gc/mL) and AAV1/2-CAG-FLEX-tdTomato (MPI in house production, 3.5 × 10^12^ vg/mL) at a volume ratio of 24:6:1 (titer ratio: 14.4:3.9:5.8). The plasmid for hSyn-DIO-GFPNtgephyrin AAV production was a kind gift of Dr. Pablo Mendez, Cajal institute, Madrid, Spain. Hippocampal window implantations were performed according to standard procedures^103^ and local regulations. Briefly, mice were anesthetized with isoflurane (2.5% to 1.5% in air) and injected subcutaneously with Meloxicam 2 mg/kg. An incision of the scalp was performed, the skull cleaned of the periosteum and a craniotomy was performed using a dental drill equipped with a trephine (∅ 3.5 mm) centered on the AP and ML coordinates of the AAV injection. After removing the bone flap, the cortex above the dCA1 was removed by suction—under constant flushing with cold saline—leaving the alveus fibers intact. A stainless-steel cylindrical window capped by a cover slip (∅, 3.5 mm; height, 1.65 mm + 0.13–0.16 mm cover slip) was then inserted above the alveus and glued to the skull with a three-component dental cement (Super Bond, Sunmedical). A head holding aluminum plate was then positioned at an angle of 90 degrees to the window roughly and glued with Kallocryl A/C dental cement (Speiko). 2 P in vivo imaging started after a minimum of 2 weeks recovery.

### Trace fear conditioning

On the day of training (D6), mice were placed into a conditioning chamber (19 cm × 19 cm), metal grid floor, white light illumination, and ethanol odor (Context A) in a sound isolated box (Panlab, Barcelona, Spain). After 3 min of habituation, mice received 4 mild electric foot shocks (0.75 mA, 1 s duration, US) paired to a tone (9 kHz, 20 s duration, CS) with a trace of 20 s between the tone and the shock and an intertrial interval of 105 s. On D12, mice were placed into Context A for 3 min and later, mice were put into a novel Context B (15 cm diameter round transparent chamber with bedding, white light illumination, and acetic acid odor). In this new Context B, the CS tone was played for 1 min, after 3 min of habituation. The position of the mouse was tracked, and the freezing behavior was monitored and quantified with ANY-maze software (Stoelting, Wood Dale, IL, USA). The freezing response was calculated as the percentage of time during which the mice were not moving over the total time of probing the memory.

### Tissue preparation and immunohistochemistry

Mice were intracardially perfused with ice-cold 1x phosphate-buffered saline (PBS), followed by 4% paraformaldehyde (PFA) in PBS. Next, brains were collected and post-fixed in 4% PFA-PBS for 24 h at 4 °C and then transferred to 30% sucrose in PBS for 48 h at 4 °C. 40 μm thick brain slices were cut with a vibratome (Thermo Fisher Scientific, Microm or Leica) and stored in 1x PBS containing 0.025% Sodium Azide. Prior to staining slices were first incubated in 150 mM glycine in PBS for 15–30 min at room temperature, followed by three 5 min-washes in PBS. Slices were then incubated in blocking buffer (10% normal goat serum, 0.8% Triton X-100 in PBS) for 1–2 h at room temperature, followed by three 5 min-washes in PBS. Slices were then incubated with AnkyrinG primary antibody (Synaptic Systems #386006) diluted 1:500 in antibody buffer (5% goat serum, 0.4% Triton X-100 in PBS) at 4 °C, followed by three 5 min-washes in PBS. Slices were then incubated in secondary Alexa 405 antibody (Abcam ab175675) diluted 1:500 in antibody buffer at 4 °C, followed by three 10 min-washes in PBS. 20 µm thick brain slices were cut using a cryostat (Leica CM3050S) after embedding fixed brains in OCT compound (Leica Tissue Freezing Compound). Slices were then blocked in 5% BSA in 0.2% Triton-PBS (1 h at room temperature), vGAT (Synaptic Systems #131308) diluted 1:200 in staining solution (2.5% BSA, 0.2% Triton in PBS) and incubated at 4 °C, followed by 3 washes in staining solution and finally incubated with secondary antibodies (IgG ATTO647N (H + L), Hypermol #2710-1MG) diluted 1:500 in staining solution for 2 h at room temperature. All antibody incubations lasted 22 h (unless specified) and were performed under mild shaking. After staining, slices were mounted on Super Frost plus glass slides (Thermo) and allowed to dry protected from light. Once dry, the samples were covered with either Mowiol or Vectashield Hard Set mounting medium, and a cover glass was placed on top. The slides were left to dry for at least 4 h or overnight under cover, and were subsequently stored at 4 °C.

### Confocal imaging

Confocal Images were acquired using a Leica TCS SP8 X confocal microscope equipped with a Leica HC PL APO 63x/1.40 OIL CS2 oil-immersion objective (2048 × 2048 pixel sampling, 0.0225359 × 0.0225359 × 0.2498629 µm^3^/voxel)

### In vivo imaging

Longitudinal 2 P imaging was performed in anesthetized mice during the light phase every 48 h for 13 days. Each mouse underwent a previous imaging session to acquire overview images and determine appropriate laser power levels per image which were then maintained through time (range of 30–60 mW). Animals were placed on a 37 °C heating pad for the duration of each imaging session and eyes were covered with Bepanthen eye ointment. Each mouse head was manually aligned to the light path as previously described^103,104^ prior to each imaging session. Mice were imaged using either a Bruker Ultima IV microscope—equipped with an InSight Deep See dual color laser system (Spectra-Physics), a 1.0 NA, 25x water immersion objective (Olympus XLPlan N 25x/1.00 SVMP) and a galvanometric scanner—or a Thorlabs Bergamo microscope—equipped with a Chameleon Ti:Sapphire dual color laser system (Coherent), a 0.8 NA 40x water immersion objective (Nikon NIR Apo 40x/0.80 W DIC N2) and a resonant scanner-. TdTomato and GFP were co-excited using 1040 nm and 920 nm wavelengths and their fluorescence signals were split in two in the emission path using filters and detected by two GaAsPs (Hamamatsu or Thorlabs 2102/M). 3D stacks were acquired at 512 × 512 pixel width (0.094 × 0.094 × 1 µm^3^/voxel size, 50–100 z-planes, 20 repetitions per each z-plane at the Bruker system or 0.1079 × 0.1079 × 1 µm^3^/voxel size, 50–100 z-planes, 15 repetitions per each z-plane at the Thorlabs system). Filter sets used to split the two channels were MDF-GFP (Thorlabs), et750sp-2p_t560lpxr-UF1, and t570lpxr-UF1(Chroma).

### Processing of 2 P image stacks

First, each z-plane that was repeatedly acquired was registered to the mean of all repetitions of that z-plane with a Rigid Body registration and then averaged to a single plane. For 2 channel images the red channel (cytosolic tdTomato) was used for reference and the same transformations were then applied to the green channel. Next, all registered and averaged z-planes were concatenated to a 3D image stack for deconvolution. 3D blind deconvolution was performed using AutoQuantX3 software and 10 iterations per stack. We then semi-automatically equalized the z-dimension in size and section of all time point 3D image stacks to concatenate them into an ImageJ timeseries 4D stack. To correct for motion artifacts in time we used a registration based on cross correlation. For 2 channel images, the red channel was used as registration reference.

### Tracking of synapses and their size over time

Prior to synaptic tracking the experimenter was blinded to the experimental groups. To track IS and spines on 4D timeseries image stacks we used the ImageJ Plugin, TRACKMATE^105^. Each synapse was detected at the plane where it had its maximum diameter and it was assigned a unique identifier (ID) that included x, y, and z positional coordinates for the synapse. When a synapse disappeared and re-appeared at what appeared to be the same location, we assigned a new ID.

To calculate the area of ISs, we defined a circular region of interest (ROI) for each identifier and adjusted the size of the ROI to roughly match the size of the visible GFP signal. We then defined all pixels in the ROI whose intensity was above a threshold obtained by using Otsu’s method as belonging to an IS. Finally, we multiplied this value for the pixel size to obtain the IS area.

To calculate the areas of dendritic spines, we segmented single dendritic spines and then matched these segmented spines to the tracking data. Tracked locations were further refined using iterative closest point based metric^106^. To segment dendritic spines, we implemented a convolutional neural network (CNN) trained on a large-scale dendritic spine dataset from a previous study^107^ and fine-tuned with additional annotations from this in vivo dataset. This allowed the network to generalize across different imaging conditions and accurately segment spines and dendrites in both in-vitro and in vivo datasets. The segmentation involved extracting localized image patches centered on the manually tracked spine locations, which were then passed through the CNN. The network output consisted of a probability map highlighting spines and dendrites, which was thresholded and converted into a binary segmentation mask. To further refine the segmentation and enhance dendritic visibility, we applied a fibermetric-based enhancement filter optimized for bright, elongated structures corresponding to dendrites and spines. This method is based on a Hessian-derived multiscale Frangi vesselness filter. The fibermetric function does not classify structures but instead enhances their visibility, making them more distinguishable from the background, which is particularly useful for dendrites and spines, as it highlights their tubular morphology while suppressing non-relevant background features. This was followed by anisotropic diffusion filtering, which reduced noise while preserving fine morphological features. After contrast adjustment, an ROI—including both the dendritic shaft and the spine head—was extracted for each spine. We measured spine volume through the integration of fluorescence intensity within the segmented spine regions, with local background subtraction applied to improve accuracy. The CNN-generated segmentation was assigned a confidence score based on classification probability, providing a reliability measure for each detected spine. Overlay images were generated to display the segmented spines on the original images, allowing for direct comparison between tracked and segmented spines. In addition to segmentation confidence scores, each segmentation was manually assigned a quality control (QC) score to validate segmentation quality (1 = segmented; 0 = not segmented well) and if a single synapse within a synaptic site was assigned a score of 0, the entire site was excluded from the QC dataset. Only spines that passed manual curation for volume segmentation were included in volume analysis, all spines were included in survival analysis.

Temporal changes in spine morphology were visualized across time points, and the final dataset included spine volume, area, dendritic fluorescence intensity, and segmentation confidence scores, all of which were exported for statistical analysis.

### Statistics and reproducibility

No statistical method was used to predetermine sample size. Mice were independent biological replicates; neurons, dendrites, and synapses were nested biological observations, and longitudinal imaging days and intervals were repeated measurements. Exact sample sizes for individual analyses are reported in the figure legends and Source Data. Mice were randomly assigned to control or TFC groups before the start of the experiments. The experimenter performing synapse tracking was blinded to group allocation. No animals were excluded from the analyses. In some analyses, data were excluded only according to the quality-control and minimum-count criteria described in the relevant analysis sections below.

All statistical analysis was performed in R (version 4.4.1). Due to the nested structure of the data, mixed-effects models were used. Frequentist models were applied for size comparisons with continuous outcomes and relatively simple random-effects structures using the nlme package (version 3.1.164). For inference from frequentist models, CR2 cluster-robust variance estimation from the clubSandwich package (version 0.6.0) was used, with clustering at the mouse level and small-sample correction for hypothesis testing. Statistical significance was assessed at *p* < 0.05. For statistical comparisons among three groups, multiple testing was controlled for using a closed-testing procedure: an omnibus test was performed first and, following rejection of the omnibus null hypothesis, the three pairwise hypotheses were tested, with adjusted *p* values defined as the maximum of the omnibus and corresponding pairwise *p* values. *p* values for all contrasts are reported in the Source Data file.

Bayesian models were used for longitudinal synapse number, survival, and turnover analyses using the brms package (version 2.22.0), as these analyses involved interval-censored outcomes, distributional regression, and complex random-effects structures that are not jointly supported by standard frequentist mixed-model packages. Posterior distributions were estimated using Markov chain Monte Carlo sampling. Weakly informative priors were used. Prior sensitivity checks confirmed that posteriors of fixed effects were dominated by the data rather than the prior. Model fit was evaluated using posterior predictive checks. Convergence and sampling efficiency were assessed using trace plots, potential scale reduction factors (R̂), and effective sample sizes. For Bayesian models, evidence for fixed effects was quantified using the probability of direction (pd). Throughout the paper, effects with pd >0.95 were considered statistically significant. All contrasts with credible intervals (CrIs) and pds are shown together with the raw data in the Source Data file. AnkyrinG stainings were repeated three times and Ankyrin + vGAT stainings were repeated two times with similar results.

### Synaptic size comparisons

Compartment-specific (or dendritic order–specific) differences in synapse size across days were analyzed using frequentist linear mixed-effects models (LMMs). ISs and spines were analyzed in separate models. For spines only volumes that passed a manual curation for correct segmentation were used for inference. Synapse size was analyzed on the logarithmic scale to satisfy normality assumptions. Compartment (or dendritic order) was included as a fixed effect. The hierarchical structure of the data was modeled using nested random intercepts for individual compartments (dendrites, DOs) within neurons within mice. To assess size differences related to synapse stability, synapses were classified as transient (observed on only one day) or persistent (present at all measured time points). For persistent synapses, the mean size across all days was used, whereas for transient synapses the single-day measurement was retained. Because persistent synapses were represented by averages across multiple days whereas transient synapses contributed single-day measurements, the model allowed for different residual variances between stability groups. Since substantial variability in the persistent–transient size difference of ISs was detected across neurons and compartments, a random slope for stability was additionally included at the compartment-within-neuron-within-mouse level.

To assess the maintenance of synaptic size hierarchies over time, Kendall’s rank correlation coefficient (*τ*) was computed between Day 1 and subsequent time points for individual neurons. This non-parametric measure was used to quantify the ordinal association of synapse sizes and is robust to global scaling and non-linear growth effects. Kendall’s τ was chosen over alternative rank-based metrics due to its favorable statistical properties in neurons with low synaptic counts. Neurons retaining fewer than four paired synapses at any comparison time point were excluded to ensure stable correlation estimates.

### Number of synapses per neuron over time

Longitudinal changes in synaptic density were analyzed using Bayesian generalized linear mixed-effects models. Separate models were used for ISs and spines. Synaptic counts per neuron were modeled using a negative binomial distribution with a logarithmic link function to account for potential overdispersion in count data. Weakly informative priors were used for regression coefficients and variance parameters. The hierarchical structure of the data was modeled using nested random intercepts for compartments within neurons within mice (neurons within mice for spines models) to account for repeated measurements and the nested sampling design. To allow for variability in longitudinal trajectories across animals, compartment-specific random slopes for Day were additionally included at the mouse level. To characterize baseline dynamics across compartments (Fig. 3b/ S2d), synaptic counts were modeled as a function of the interaction between Day and Compartment (or just Day in spines models). To evaluate potential TFC-induced changes in synaptic number (Fig. 7c,f,i,l /S5,i.,j) different from baseline, the models were extended to include an interaction between Group (experiment vs control) as well, where Group indicated whether measurements originated from the TFC experiment or the baseline imaging paradigm. Day-specific contrasts in the TFC group were only marked as significant if both the pd of the comparison within the TFC group as well as the pd of the interaction with the control null reference was above 0.95. For visualization, population-level posterior predictions were generated on the response scale and summarized as posterior medians and 95% credible intervals of the expected synapse count per neuron.

### Baseline survival dynamics

Posterior distributions of synapse lifetimes were modeled using log-normal Bayesian accelerated failure time (AFT) models, to compare synaptic stability of ISs across compartments, between dendritic ISs and spines, as well as populations (newborn vs pre-existing synapses). The log-normal AFT formulation permits hazard rates to be dependent on synapse age, consistent with increased stability of longer-lived synapses. The synapses’ birth, death and the censoring of measurement at day 13, as well as the interval-censored nature of the data—arising from discrete imaging at two-day intervals—was explicitly incorporated into the likelihood. Separate models were fitted for spines, ISs and the comparison between spines and dendritic ISs. Population (newborn versus present at Day 1) and—for ISs—Compartment (including their interaction) were included as fixed effects. The hierarchical structure of the data was accounted for by including random intercepts for compartment nested within neuron nested within mouse. To obtain an interpretable summary measure of synaptic stability for statistical comparison across synapse types, populations, and compartments, the restricted mean survival time (RMST) was obtained by numerically integrating the posterior survival functions. Pairwise differences in RMST were assessed using posterior contrasts.

Separate Bayesian log-normal AFT models were fitted for compartments and spines, including baseline synapse size as a fixed effect, to investigate the relationship between synapse size and synapse survival. For spines only measurements that passed a manual curation for correct volume segmentation were used for inference. RMST was estimated for synapses with baseline sizes corresponding to the 25th and 75th percentiles of the observed size distribution, and pairwise differences in RMST were assessed using posterior contrasts. For visualization of dendritic IS stability across DOs, DO was additionally included as a fixed effect and dendrite id as a nested random effect. Predicted RMST values were calculated under size-standardized conditions in which synapse size was fixed to the median of the respective dataset, allowing the investigation of size-independent contributions of dendritic order to synapse stability.

### TFC effects on synaptic turnover dynamics

Turnover was modeled as a binomial process in which gains or losses were expressed relative to the total number of synapse observations across both timepoints of each 48-h interval. The binomial Bayesian mixed-effects models also naturally account for the fact that neurons with fewer synapses carry more uncertainty, so their estimates are weighted accordingly. Separate models were used for ISs and for spines. For ISs the model specified a three-way interaction between Group (TFC group vs. Control group), Day Interval, and Compartment and for spines it specified a two-way Group x Interval interaction as fixed effects. The hierarchical structure of the data was accounted for by including random intercepts for compartments (in ISs) nested in neurons nested in mice. Additionally, random slopes for day intervals were modeled on the mouse and neuron levels (separately for compartments for ISs) to account for heterogeneity of mouse reaction on TFC, as well as for the heterogeneity of the reaction of different neurons within the same mice to TFC and to reduce the risk that estimated population effects are dominated by a few outlier mice or neurons. An observation-level random intercept was additionally included to account for potential overdispersion (extra-binomial variation). Since no significant baseline drift was observed in ISs, specific experimental epochs were defined by aggregating imaging intervals to generate stable reference estimates: Baseline (Day 1-Day 3 and Day 3-Day 5), TFC (Day 5–Day 7), Post (Day 7–Day 9 and Day 9–Day 11), and the Recall (Day 11–Day 13). For all epochs involving aggregation, averaging was performed directly on the posterior draws of the fitted model.

48 h and 96 h survival was modeled as a binomial process in which losses were expressed relative to the corresponding number of synapses available at the start of the respective interval. Separate models were used for 48 h and 96 h intervals, as well as for ISs and spines. For ISs the models specified a three-way interaction between Group (TFC group vs. Control group), day Interval, and Compartment and for spines it specified a two-way Group x Interval interaction as fixed effects. The hierarchical structure of the data was accounted for by including random intercepts for compartments (in ISs) nested in neurons nested in mice. Additionally, random slopes for day intervals were modeled on the mouse and neuron levels (separately for compartments for ISs) to account for heterogeneity in mouse-specific responses to TFC, as well as for the heterogeneity of the reaction of different neurons within the same mice on TFC and to reduce the risk that estimated population effects are dominated by a few outlier mice or neurons.

In all TFC-related analyses, the control group served as the primary null reference to distinguish specific TFC induced effects from common ongoing background drift in turnover stemming from the experiments’ longitudinal imaging design. Interval-specific contrasts in the TFC group were only marked as significant if both the pd of the comparison within the TFC group as well as the pd of the interaction with the control null reference was above 0.95.

### Modeling and simulations for the artificial neuronal network

All simulations were carried out by using the CoBeL-RL simulation framework using the Tensorflow machine learning library^108^. The simulations consisted of a virtual agent, navigating a virtual environment. The virtual agent was controlled by an artificial recurrent neural network implementing the hippocampal microcircuit model. The artificial agent received visual feedback in the form of naturalistic images generated using Blender 3D computer graphics software (version 2.79). The virtual environment was a squared 2 by 2 meters box, the walls of which were structured with photo-realistic textures. A point light source with adjustable color was placed above the center of the environment box. For the standard, baseline environment, the box was lit with white light and it contained a grid of 4 × 4 possible agent positions. The agent could freely move between these positions by generating actions. Images were captured at the agent’s current position using wide angle camera objects, giving a field of view of ca. 240°. Inputs corresponded to the rendered camera images scaled to 12  × 20 pixels (with 3 color channels each), giving a total of 12 × 20 × 3 = 720 visual input channels. Inputs were presented to the network by setting the activations of the 720 visual input neurons to the current values of the input channels.

### Artificial recurrent neural network implementing the hippocampal microcircuit model

The network consisted of 1008 excitatory inputs, 550 excitatory outputs, and 100 modeled inhibitory neurons (INs). Excitatory input neurons were divided in three subpopulations: 720 visual, 144 sound and 144 shock neurons. Inputs were presented to the network by setting the activations of the neurons to the current values of the input channels. Sound and shock neurons were only active during TFC, otherwise they were silent throughout the experiment. All excitatory input neurons projected to the dendritic compartment of all excitatory output neurons. Excitatory output neurons consisted of 3 compartments (dendrite, soma, AIS). Each compartment received input projections from a distinct set of inputs (excitatory and/or inhibitory, see Fig. 8a) and a Rectified Linear Unit (ReLU) non-linearity was applied to generate the compartment output. The 550 outputs of the axon compartment projected to (i) 4 neurons that controlled actions of the 4 different actions of the agent to move within the maze (up, down, left, right) (ii) to INs that synapsed on the AIS of the output neurons (iii) to a set of INs that inhibited soma-projecting INs and (iv) recurrently to their own dendritic compartment. INs belonged to 4 subpopulations with connectivity and relative abundance designed to recapitulate the local connectivity of the mouse dCA1 hippocampus. INs were single compartment neuron models with ReLU activation to produce the output. All INs and excitatory compartments used adaptive bias weights that were included in the training process. Synaptic weights were constrained to being either excitatory (positive) or inhibitory (negative). To prevent synapses from switching signs and to model synaptic rewiring, we adopted the method from^109^. Briefly, for every synapse *i* we introduced a synaptic parameter *θ*_*i*_ which was projected using a non-linear mapping *w*_*i*_ = exp(T*(*θ*_*i*_ – *θ*_0_)) > 0, where we chose the temperature parameter T = 0.25 and offset *θ*_0_ = 1. Synapses with *θ*_*i*_ ≤ 0 were interpreted as disconnected synapses and their effect on the post-synaptic neuron was set to zero. A single synapse was allowed between any pair of neurons in the circuit to simplify the simulation.

### Simulation of the agent and TFC

The agent was trained using the deep Q-learning algorithm. Briefly, the hippocampal network model was trained to approximate the action-value function, which represents the expected time-discounted reward for given state / action pairs. We used a discount factor of 0.8. Weight updates were superimposed by random Gaussian noise with amplitude eta (η) to facilitate synaptic motility. The discount factor depreciates the value of rewards far in the future and thus encourages fast goal seeking. States were not presented explicitly (e.g. as locations in the maze), but only implicitly through visual, sound and shock inputs. One of 16 positions in a standard environment (context A—a squared box, lit with white light with walls textured with a simple brick pattern) was randomly selected as reward location. Whenever the agent visited the reward location, a reward of +10 was delivered to the agent and the reward was immediately relocated to a new random position. The agent resided for 4500 time steps in context A. Connectivity dynamics were recorded during this period and labeled as Baseline. The agent was then placed in a novel context for 450 time steps. In this context (context B), a red light source was used and walls were textured with different patterns. The TFC environment had otherwise the same geometry as the context A. Textures were selected to be easily distinguishable also in low-resolution images but had otherwise no significance. In this context, no reward was provided to the agent. After an initial delay of 90 time steps, the first TFC trial was initiated. Every TFC trial had a total duration of 90 time steps. The sound neurons were activated for 10 time steps, followed by a delay of 10 time steps. Then the shock neurons were activated for 1 time step, accompanied by a strong negative reward of −100, irrespective of the agent’s current location. After the shock a delay of 69 time steps, where shock and sound neurons were inactive, was provided. TFC trials were repeated 4 times before the agent was transferred back to context A. The activation values of sound and shock neurons were set to 1 during TFC trials, but they were silent throughout the rest of the experiment.

### Analysis of the circuit re-wiring

We recorded synaptic parameter values in intervals of 90 time steps throughout the whole experiment to evaluate IS turnover and the rewiring of the circuit. Synapses were labeled as active if their synaptic parameter *θ*_*i*_ was >0 and as inactive otherwise. The calculation of the surviving fraction was performed as for in vivo experiments. Synapses present after trial 1 (time step 90) were used as ISs. Present D1 synapses in Fig.8d. Noise amplitudes *η* were used to calibrate the behavior of the circuit, i.e., to qualitatively match the in vivo behavior for the baseline condition (*η* = 0.01 for dendrite, *η* = 0.2 for soma, and *η* = 0.08 for AIS ISs). After calibrating to reproduce baseline behavior, noise amplitudes were kept fixed during the whole experiment and for further evaluations, including the TFC analysis.

### Reporting summary

Further information on research design is available in the Nature Portfolio Reporting Summary linked to this article.

## Supplementary information

Supplementary Information

Reporting Summary

Transparent Peer Review file

## Source data

Source Data

## Data Availability

Source data are provided with this paper as Excel document containing all numerical values needed to generate each plot or table. Due to the size of the raw images, they will be provided without restriction upon request. Point of contact for data availability is Alessio Attardo. Source data are provided with this paper.

## Code availability

The code has been uploaded into Code Ocean (DOI link: 10.24433/CO.9762429.v1) and, additionally, will be made available upon request. Points of contact for code availability are: Felix Kuhn and David Kappel.
