# Supplementary material for: Learning reorganizes dendritic and stabilizes axon initial segment inhibitory synapses in CA1 pyramidal neurons
Source: Nat Commun. 2026 Sep 14;17:9791. doi: 10.1038/s41467-026-77800-w (PMC13575122; doi:10.1038/s41467-026-77800-w)
Supplement: Supplementary file 1 [file 41467_2026_77800_MOESM1_ESM.pdf]

Learning reorganizes dendritic and stabilizes axon initial segment inhibitory synapses in CA1 pyramidal neurons

Hannah Klimmt [1,2§], Felix Kuhn [1§], David Kappel [3], Bhargavi Murthy [1,4], Alessandro Francesco Ulivi [1], Ali Öztürk Argunşah [5], Silvia Vieweg [1], Rosa Eva Huettl [6], Stefan Remy [1,7,8] and Alessio Attardo [1,7*]

1 Leibniz Institute for Neurobiology, Magdeburg, Germany

2 International Max Plank Research School for Translational Psychiatry, Munich, Germany

3 Center for Cognitive Interaction Technology, Bielefeld University, Bielefeld, Germany

4 Graduate School for Systemic Neuroscience, Ludwig-Maximilian University, Munich, Germany

5 Brain Research Institute (HiFo), University of Zurich and Neuroscience Center Zurich, Zurich, Switzerland.

6 Max Planck Institute of Psychiatry, Munich, Germany

7 German Center for Mental Health (DZPG), partner site Halle-Jena-Magdeburg and Center for Behavioral Brain Science (CBBS), Magdeburg, Germany

8 German Center for Neurodegenerative Diseases (DZNE), Magdeburg, Germany

§ These authors equally contributed to this work

* Corresponding author: alessio.attardo@lin-magdeburg.de

Supplementary figures and captions

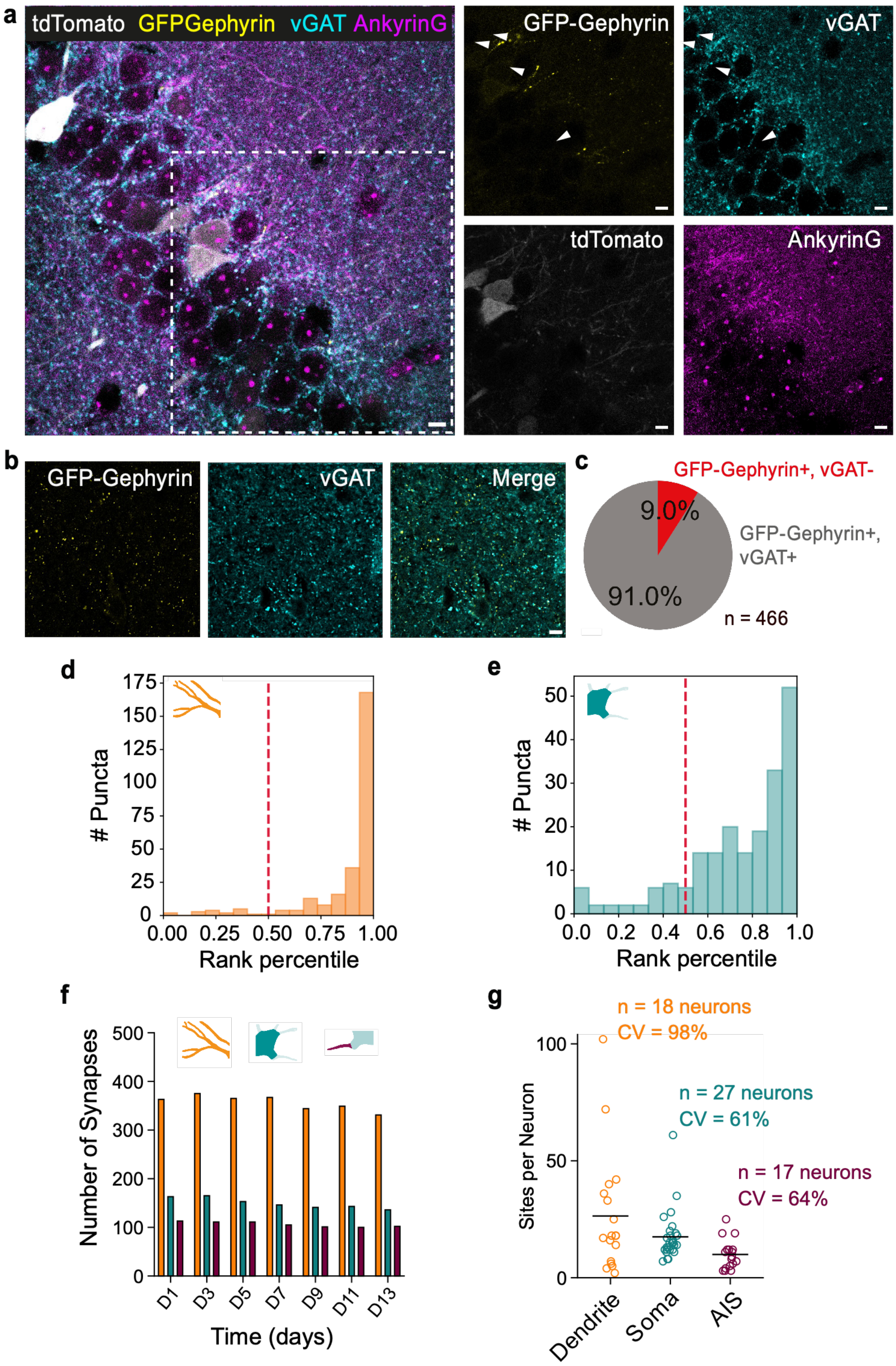

Figure S1 | Colocalization of Gephyrin-GFP with inhibitory synaptic markers and numbers of inhibitory synapses and synaptic sites.

a, confocal images (MIP of 3 slices, z-step 0.29 µm) of hippocampal CA1 pyramidal cell layer. Left: merge of all 4 channels (tdTomato, white; GFP-Gephyrin, yellow; vGAT, cyan; AnkyrinG, magenta), white square indicates ROI in higher magnification on the right. Right: single channels of ROI zoomed in, arrowheads point to vGAT positive Gephyrin puncta. Scalebars 5 µm.

b, deconvolved single confocal Z planes of hippocampal CA1 S. Oriens (GFP-Gephyrin, yellow; vGAT, cyan). Scale bars 5 µm.

c, synapses counted from 3 images (Gephyrin-GFP puncta = 466, Gephyrin-GFP-positive, vGAT-negative puncta = 42, Gephyrin-GFP-positive, vGAT-positive puncta = 424).

d, Gephyrin puncta of the basal dendritic layer were reanalyzed using a local shifted-channel control to estimate chance levels of VGAT association. For each punctum, the observed VGAT apposition score was defined as the 95th percentile of Gaussian-smoothed VGAT intensity within the corresponding Gephyrin mask and was ranked relative to 100 randomly shifted-channel controls generated by 2–3 µm x-y displacements. Rank distribution was shifted clearly above chance level indicated by the red dashed line (median rank = 0.97; n= 267 puncta).

e, same analysis as in **d**, shown for Gephyrin puncta in the somatic layer (median rank = 0.82; n=199 puncta).

f, number of dendritic (orange), somatic (teal) and AIS (purple) (356, 150 and 106, average per day; 18, 27, 17 neurons respectively, 6 mice) ISs per imaging day during baseline.

g, number of dendritic (orange), somatic (teal) and AIS (purple) inhibitory synaptic sites per neuron during baseline.

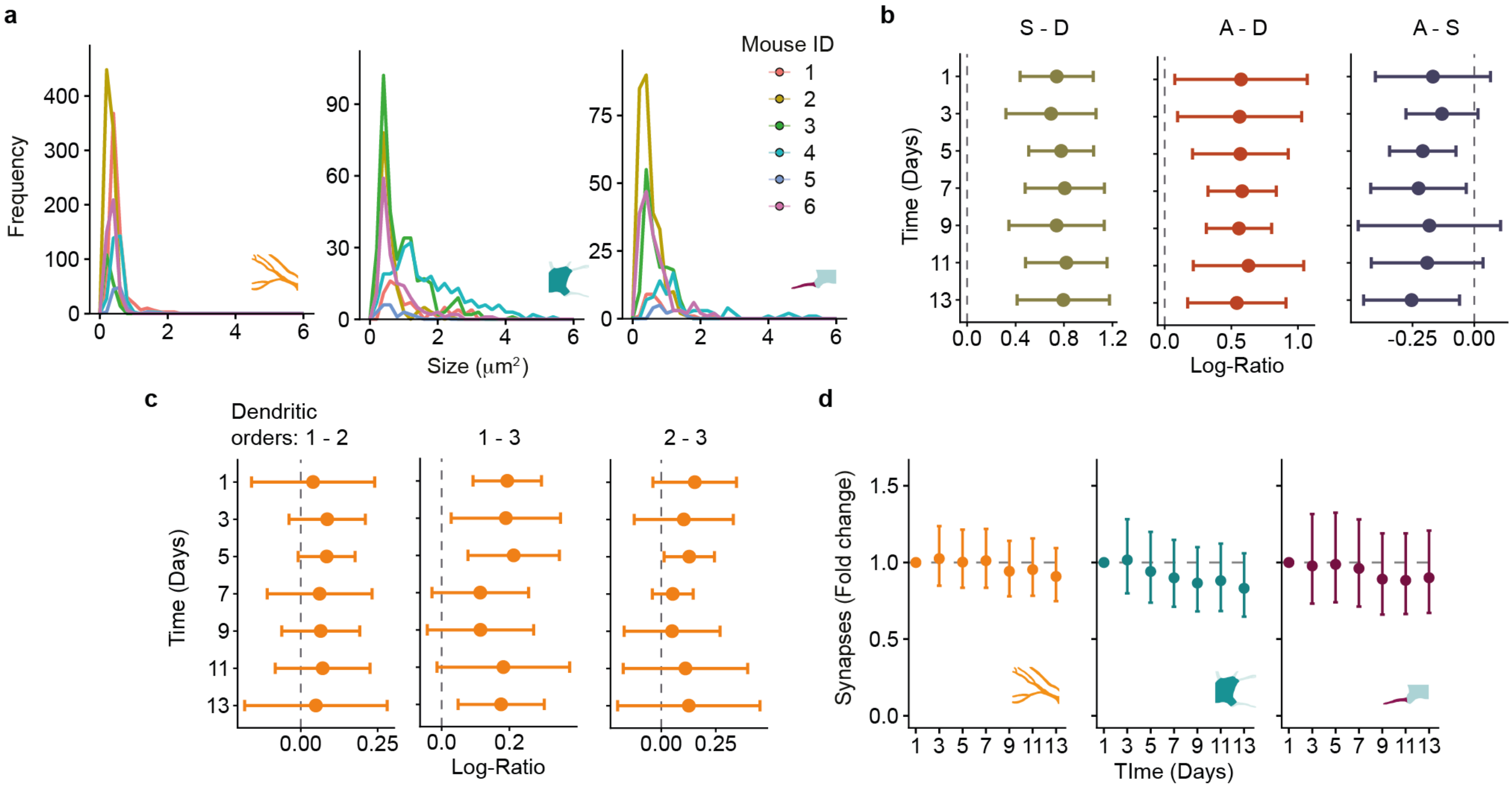

Figure S2 | inhibitory synapses sizes and counts through time during baseline.

a, frequency distribution of ISs volumes per 0.2 μm^2 across compartments colored by individual mice.

b, forest plots showing log-ratios of mean ISs size grouped by compartments across recording days. Points and horizontal bars represent estimates with 95 percent CIs from linear mixed-effects models with nested random intercepts (mouse/neuron/compartment) and CR2-robust standard errors.

c, same analysis as in **(b)** but for dendritic ISs only grouped by dendritic order.

d, median posterior fold change in spine number per neuron of each measurement day versus day 1, grouped by compartment (basal dendritic, orange; somatic, teal; AIS, purple). Values derived from a Bayesian negative binomial model with nested random intercepts for mouse, neuron and compartment. Dots show posterior medians with 95% credible intervals (CrIs).

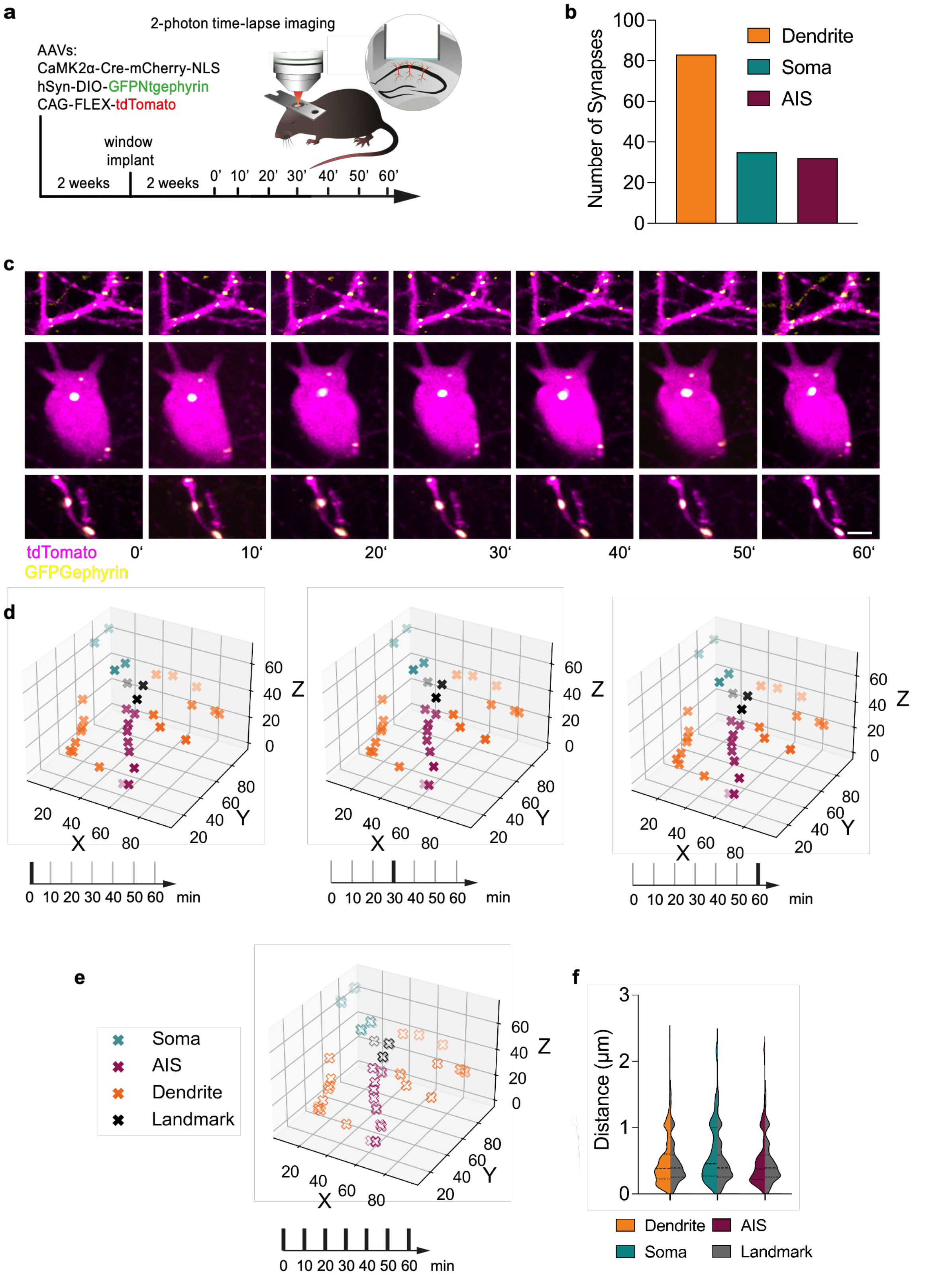

Figure S3 | Three-dimensional euclidean distances of stable inhibitory synapses at short time intervals

a, schematic description of the strategy to label a sparse subset of dCA1 PNs and the ISs impinging on them as well as of the experimental timeline. Illustration adapted from Chenani et al. (2022), CC BY 4.0.

b, numbers of stable dendritic, somatic, AIS inhibitory sites (83, 5 and 32 respectively).

c, two-photon time series of dCA1 PN subcellular compartments (dendrites, top; somata, middle; axon initial segments, bottom) expressing GFP-Gephyrin (yellow) and dTomato (magenta). Scale bar, 5 µm.

d, centroid coordinates of dendritic (orange), somatic (teal) and AIS (purple) synapses and 3 structural landmarks (black) of example neuron plotted in 3D vector space of 3 timepoints.

e, overlay of dendritic (orange), somatic (teal) and AIS (purple) synapse centroid coordinates and 3 landmarks (black) of example neuron plotted in 3D vector space of 6 timepoints.

f, distributions of euclidean 3D distances between adjacent timepoints of the same synapse centroid or landmark. (Landmark vs. Dendrite $p > 0.9999$; Landmark vs. Soma $p = 0.1287$; Landmark vs. AIS $p > 0.9999$; Kruskal–Wallis test followed by two-sided Dunn's multiple-comparisons tests; $n_{Dendrite} = 470$; $n_{Soma} = 167$; $n_{AIS} = 66$ distances; $n_{Landmark} = 66$)

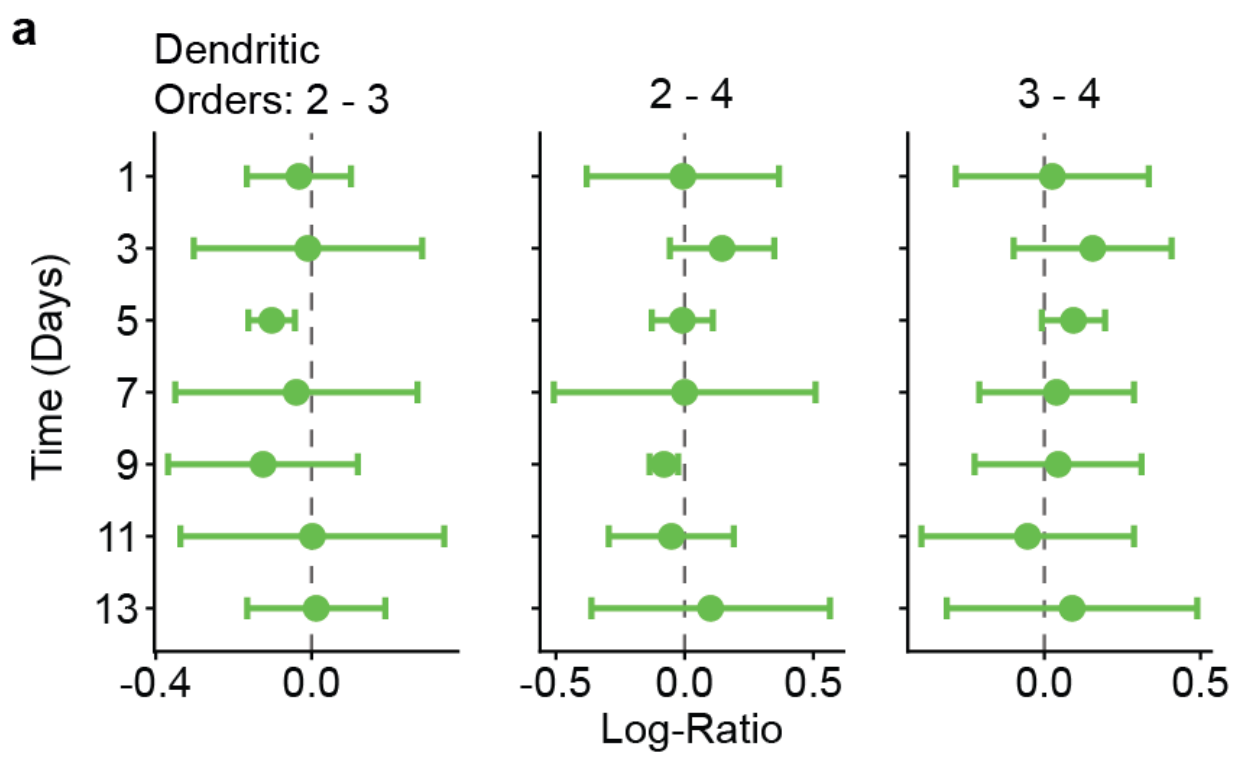

Figure S4 | 48h Turnover and stability of inhibitory synapses and dendritic spines in TFC group.

a, forest plots showing log-ratios of mean spine size between dendritic order pairs across recording days. Points and horizontal bars represent estimates and 95% CIs from linear mixed-effects models with nested random intercepts (mouse/neuron/dendrite/order) and CR2-robust standard errors. Dashed line indicates no difference.

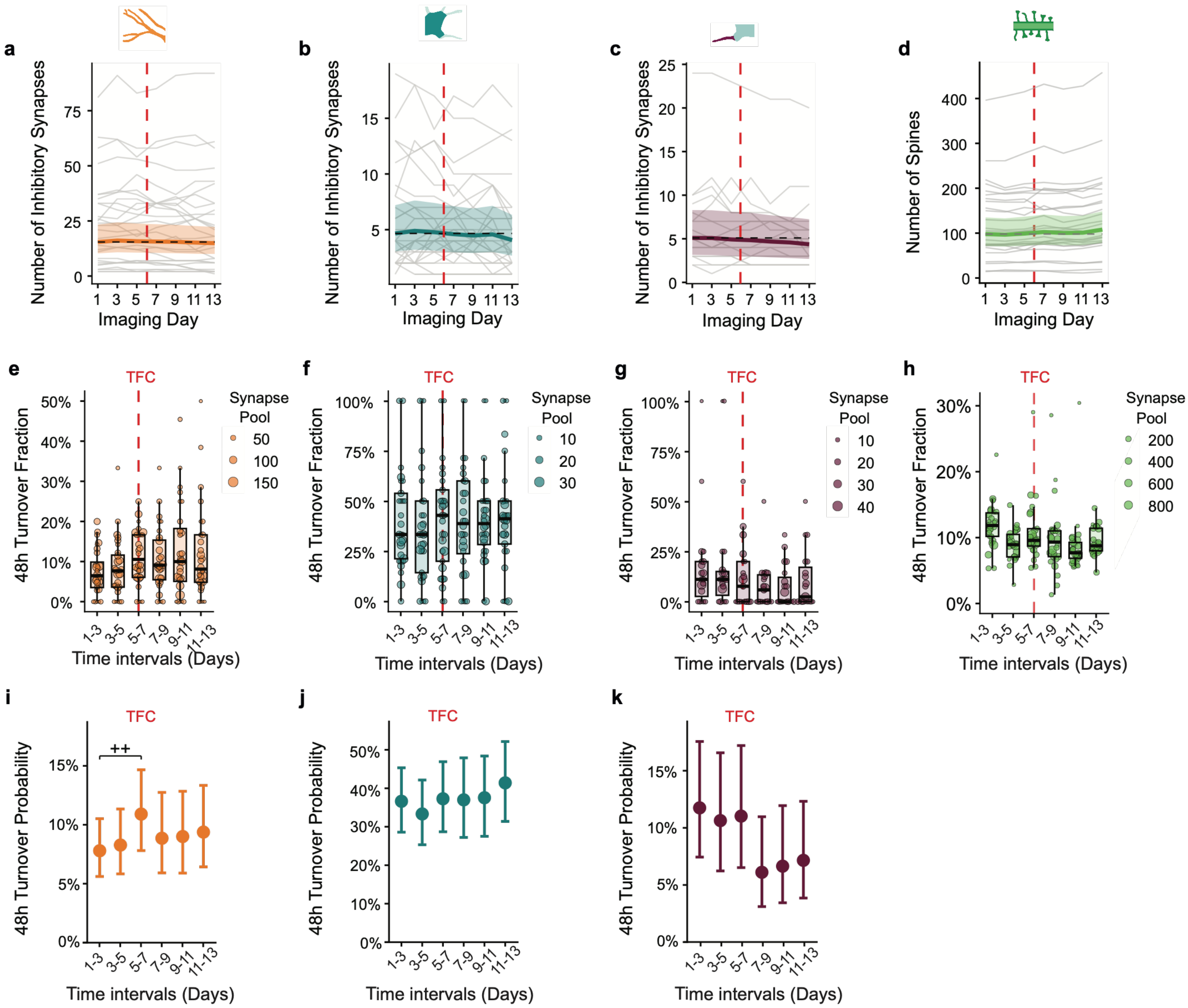

Figure S5 | Synaptic dynamics upon learning a fear association.

a-d, ISs counts per neuron across days across TFC cohort; (**a**) basal dendritic ISs, (**b**) somatic ISs (**c**) AIS ISs (**d**) dendritic spines. Thin lines show individual neurons; thick lines show model-estimated population medians from a Bayesian negative binomial model with nested random intercepts for mouse, neuron, and in the ISs case also compartment (see Methods). Shaded areas indicate 95% CrIs; horizontal dashed lines mark the day 1 posterior median, vertical dashed lines indicate timepoint of fear stimulus; n basal dendritic ISs (mice/neurons), 8/28; somatic ISs, 8/28; AIS ISs, 7/19; basal dendritic spines, 4/22.

e-h, raw 48h turnover ratios per neuron across day intervals of the TFC group; (**e**) dendritic ISs, (**f**) somatic ISs (**g**) AIS ISs (**h**) dendritic spines. Dot sizes represent number of synapses per neuron. Boxplots show raw medians with 95 confidence intervals.

i-k, Compartment-specific 48 h turnover probabilities of TFC group. Estimates derived from a Bayesian binomial model with nested random slopes and control data (day × group interaction; see Methods). n per group as in (**a-d**). Dots show posterior medians with 95% CrIs (error bars). A significant effect was found between 48h turnover probability of Day 1 – Day 3 vs. Day 5- Day 7 in dendritic spines(Δ = 0.031, pd = 0.99^{++}, pd_interaction = 0.97)
