# Supplementary material for: Learning reorganizes dendritic and stabilizes axon initial segment inhibitory synapses in CA1 pyramidal neurons
Source: Nat Commun. 2026 Sep 14;17:9791. doi: 10.1038/s41467-026-77800-w (PMC13575122; doi:10.1038/s41467-026-77800-w)
Supplement: Supplementary file 3 [file 41467_2026_77800_MOESM3_ESM.pdf]

nature portfolio

Peer Review File

Learning reorganizes dendritic and stabilizes axon initial segment inhibitory synapses in CA1 pyramidal neurons

Corresponding Author: Dr Alessio Attardo

This file contains all reviewer reports in order by version, followed by all author rebuttals in order by version.

Version 0:

Reviewer comments:

Reviewer #1

(Remarks to the Author)
The manuscript by Klimmt et al. examines the structural plasticity of inhibitory synapses (INS) on three main subcellular domains of CA1 principal neurons (PNs): basal dendrites, somata, and axon initial segments (AIS). Using two-photon in vivo imaging, the authors monitor the size and temporal dynamics of these synapses and compare them with spines. They report that dendritic INS are less dynamic than spines during their first acquisitions, but undergo reorganization following trace fear conditioning. They also employ a mathematical model to describe the stabilization mechanisms of AIS INS.
Overall, this is an interesting paper that focuses on the plasticity of inhibitory inputs onto hippocampal PNs. However, there are several concerns, along with a significant lack of detail. Clarification of experimental methods and data analysis is necessary.
Results described in Figure 1
1. The names of AAVs are inconsistent between the figures and the Methods section.
2. The results do not emphasize that the analyzed dendritic compartment is limited to basal dendrites. This is only mentioned once in the Introduction and later in the Discussion, where the authors correctly point out previously reported differences between apical and basal dendrites. These differences relate not only to the nature of inputs but also to intrinsic properties (for example: https://doi.org/10.1371/journal.pone.0118482). In my opinion, the results should consistently specify that only basal dendrites were tracked when referring to the dendritic compartment.
3. The authors state: “AIS INS were identified by their large size—consistent with cartridge synapses—and confirmed by AnkyrinG immunostaining.” The term “large size” should be quantified and expressed in µm.
4. Maximum intensity projection (MIP) images are not ideal for identifying precise subcellular localization, particularly in panel c, where the stack is up to 40 µm thick. Given the likely overexpression due to AAV, it would be preferable to show colocalization with the cell membrane, either by rotating the 3D image or by displaying an individual optical slice where the tomato and GFP signals overlap. This applies to both Figure 1 and Figure S3 (where I assume panel c is also a MIP). The 3D centroid distance plots could also include average distances to the cell membrane.
In the same vein, staining for presynaptic markers (e.g., Neuroligin 2 or VGAT) would support the claim that the observed structures are indeed synapses.
Without such evidence, the term “putative synapses” would be more appropriate.
The same concern applies to Figure 2.
Figure 4 does not specify whether the images are stacks or single slices.
Behavioral protocols and results raise the most significant concerns:
1. You used a cued fear memory test and investigated structural plasticity in CA1. However, the amygdala is more strongly implicated in this type of memory (e.g., https://doi.org/10.1186/s13041-020-00652-y and https://doi.org/10.1037//0735-7044.106.2.274).
2. In Figure 7b, you compare Habituation A with Recall A, and Habituation B with Recall B. I assume Habituation B refers to the time in B before the tone, although this is never clearly stated. How about comparing Recall A and Recall B? Significantly lower freezing in Recall B could suggest contextual learning, thus supporting your analysis, conclusions, and the article’s title (here, the word basal should be added before dendrite). On the other hand, similar levels of freezing in both conditions would suggest an absence of learning, which would contradict the title. This cannot be assessed from the figure alone, and detailed data (e.g., averages and S.E.M. values) are not reported.
3. Additionally, the time lapse ends just one day after the recall. The remote recall phase is generally the one more dependent on hippocampal activity.
4. The inputs relevant fear context memory—if learning did occur—would primarily target apical dendrites (from the medial

entorhinal cortex), and to a lesser extent, basal dendrites.
5. Analyses concerning gain, loss and turnover of INs and spines (Figures 7c,f,i,l and S7) should compare TFC mice with control mice of Figure 3d,f. Comparing 4 days before and after training as done in Figure 7 d,g,j,m is informative but not exactly the same. The simple fact of placing mice in a different environment, under the microscope, may trigger structural plasticity mechanisms even without TFC experience.
Statistical concerns:
The authors should specify the statistical tests used for each subfigure and provide complete statistical information.
In most analyses, INS are used as the unit of analysis (n), pooling data across different time points, neurons, and animals. However, the number of animals is only reported for the behavioral experiment and in Figures S1, S4, and S6. The total number of animals is not mentioned in the Methods section. In the trace fear conditioning experiments, synapse-related data appear to disregard individual animals—or at least this is not clearly stated. Ideally, a mixed-effects model (as described in Figure 5) should be applied across all statistical analyses (e.g., https://doi.org/10.1016/j.celrep.2019.05.098). This would allow the use of larger n samples for comparisons (i.e. synapses) taking into account the variability from different animals.
Figure 6d should present dot plots.
When the authors claim that somatic INS turnover was higher than in dendritic or AIS compartments, they assert that this was not due to positional fluctuations. It would be helpful to clarify how this was demonstrated.
Minor issues in the Discussion:
1. In the paragraph “Subcellular compartmentalization of INS dynamics,” the authors mention redundancy of somatic INS compared to dendritic ones, possibly reflecting a smaller individual effect. Were multiple somatic INS significantly more abundant than dendritic INS in this study? The reported numbers suggest otherwise.
2. In “Dendritic INS are less dynamic than spines,” the phrase “Notably, the inhibitory connectivity between the basal and the apical domain…” is unclear. Did the authors mean onto both domains, rather than between them?
3. In “The relationship between size and persistency in dendritic spines and INS,” the sentence “Size decrease 48h before disappearance…” contains the word slated—this appears to be a typo and should likely be selected.

Reviewer #2

(Remarks to the Author)
The manuscript by Klimmt et al monitors structural plasticity of inhibitory synapses impinging onto pyramidal neurons in dorsal CA1 using longitudinal two-photon imaging. The authors test the hypothesis that structural plasticity is specific to sub-cellular compartments: somatic, initial axon segment and dendritic. In addition to passive recordings, the authors study the effect of hippocampal-dependent learning on synaptic changes and recapitulate the compartmentalized difference in a computational model. Structural inhibitory synapse plasticity has been studied in the cortex by a number of groups but plasticity in hippocampus has mostly been studied by one group in a series of papers detailing structural inhibitory plasticity during learning (PMID: 26807952, PMID: 24336286, PMID: 25695271). The authors report differences in stability of inhibitory synapses between compartments and also compared to excitatory synapses with compartment specific effects claimed as a result of learning. The results are therefore interesting but there are serious problems with the analysis and statistical approaches that significantly reduce confidence in the conclusions.

Major Comments
1. For statistical analysis, authors used grouped comparisons that treated individual synapses as independent data points regardless of if they were obtained from the same neuron. This would impact the variance between the somatic, AIS, dendritic defined synapses as many more dendritic INS (≤85) relative to somatic (≤20) and AIS (≤16) INS may be sampled from within the same neuron. As the activity history of an individual neuron during its lifetime would likely make these INS more similar to each other than to INS from another distinct neuron these should be accounted for. This becomes even more problematic in the longitudinal imaging studies in which an individual INS may be counted as several independent data points if present/persistent across multiple sessions which will directly influence effect size observed. Data needs to be reanalysed with mixed models that include mouse and/or neuron ID as random variables to account for this. The same issues are relevant to the analysis of spines and excitatory synapses.
2. Comparisons between putative excitatory (dendritic) and inhibitory (gephyrin) synapses are made and several conclusions drawn about differences in their overall size kinetics, however the synapses were labelled using very different methods. Dendritic labelling was measured through cell wide GFP expression in Thy-GFP mice, in comparison with more punctate inhibitory synapse labelling achieved through sparse viral labelling of inhibitory scaffolding protein expression. This introduces a large potential difference size and dynamic measurements which is not appropriately addressed. To confirm that the dynamic differences reported between excitatory synapses and INS are real and not an artifact of experimental design would need to be replicated using a similar labelling approach to GFP-Gephyrin for the excitatory synapses eg GFP-Shank.
3. The authors concede that it is not possible to distinguish individual inhibitory synapses from clusters using their imaging approach and they therefore treat clusters as individual synapses. This confounds many of the conclusions. For example, the claim is that AIS synapses are larger than other compartments but this could simply be because AIS synapse clusters contain greater numbers of synapses – which is documented. Then AIS clusters of synapses will be more stable simply because there are more synapses there. There is further circularity in the definition of AIS synapses since they are identified by having a large size.
4. The interpretations given within the discussion section are quite superficial and don’t propose many circuit or micro-circuit level connections to explain the results found. For example: “compartmentalization might stem from the specific activity patterns or genetic makeup of pre-synaptic inhibitory neurons” what kind of activity patterns in what types of inhibitory interneurons could explain the observation? “inhibitory connectivity between the basal and apical domain is very different” - how are they different? It would be better to back up the hypothesis with some specific examples.
5. The modelling of inhibitory connectivity in Fig8 assumes changes in AIS INS whereas it is well documented that somatic

and dendritic plasticity also occurs. The modelling therefore feels preliminary and simply confirms the experimental findings. To add real value the model could be used to investigate the effect of all the observed INS plasticity.

Minor comments
1. More information required on the identification and classification of AIS INS. The manuscript states “identified by large size” but requires more detail. Was the ankyrin G confirmation conducted in the subset of cases or for all AIS?
2. In Fig2 what do individual data point represent in panel e and similar for Fig 5a? Not stated and not clear.
3. What was the sampling frequency? It is stated as “as often as our technique allowed in a new set of mice” but please provide actual sampling frequency.
4. In Fig3 what does “correlation between gain and loss was higher for somatic than for dendritic and AIS INS” mean biologically? That the overall number of INS remained consistent despite higher rates of loss and gain? This is not clear. In addition, why does only somatic INS have multiple data points when legend states that each time point is represented? And how are the correlations between somatic and AIS compared statistically as there is also a sig positive correlation between loss and gain in AIS shown in panel d? It might be easier to visualise if plotted as the average lifespan of INS present at day 1 and new INS present from day 3 rather than as survival fractions.
5. In referring to Fig4 the statement “Distribution of dendritic spines’ volumes was skewed towards higher values” is unclear. There is no statistical comparison here. Does this mean higher volumes than dendritic INS or a non-gaussian distribution of size within the population.
6. In the methods much more information about surgical procedures is required including anaesthesia, analgesia, window size, aspiration fluid etc.

Reviewer #3

(Remarks to the Author)
I co-reviewed this manuscript with one of the reviewers who provided the listed reports. This is part of the Nature Communications initiative to facilitate training in peer review and to provide appropriate recognition for Early Career Researchers who co-review manuscripts.

Reviewer #4

(Remarks to the Author)
I co-reviewed this manuscript with one of the reviewers who provided the listed reports. This is part of the Nature Communications initiative to facilitate training in peer review and to provide appropriate recognition for Early Career Researchers who co-review manuscripts.

Reviewer #5

(Remarks to the Author)
In this study, the authors analyzed the structural plasticity of inhibitory and excitatory synapses onto pyramidal cells in the CA1 area of hippocampus over a period of two weeks in control mice and in mice undergoing trace fear conditioning (TFC). To image inhibitory synapses, sparse pyramidal neurons were labeled with dtTomato to visualize the structure of the neurons and gephyrin-GFP to label inhibitory synapses. For spine imaging, Thy1-GFP transgenic mice were used.. Chronic two-photon microscopy was employed to repeatedly image the same neuronal structures, allowing the quantification of synapse numbers and sizes, and their dynamics. Inhibitory synapses at dendritic branches, somata and axon initial segments (AIS) were imaged, as well as dendritic spines in a separate experiment.

The major observations are that:
-Inhibitory synapses at dendritic branches show turnover, but less so than inhibitory synapses onto the AIS. Somatic gephyrin-GFP puncta are the least stable.
-Dendritic inhibitory synapses are smaller than those on somata or AIS (though this is probably a compound labeling of multiple AIS synapses).
-Dendritic spines are more dynamic than inhibitory synapses on dendrites, but less so than gephyrin puncta at the soma.
-Inhibitory synapses at higher order dendrites are more stable.
-Dendritic spines, AIS inhibitory synapses and dendritic inhibitory synapses show a relationship between size and persistence, with smaller synapses being less persistent (though not significantly so for dendritic inhibitory synapses).
-Fear conditioning destabilizes inhibitory synapses on dendrites while it stabilizes those on the AIS. It (surprisingly) does not affect turnover of dendritic spines or somatic inhibitory synapses.
-Finally, the authors use a modeling approach to study what factors might influence AIS inhibitory synapse stability, and find that this includes the proportion of AIS targeting cells, making the AIS synapses excitatory instead of inhibitory, the influence of disinhibitory neurons and the sign of the effect (inhibitory/excitatory) of AIS synapses.

This study was a tour-de-force. The authors used challenging approaches to analyze inhibitory synapse turnover over several weeks, and developed novel tools to segment and analyze the synaptic structures. This work represents an impressive amount of work that was carried out thoroughly.
At the same time, while the findings are novel, they mostly align with what has previously been shown in neocortical areas. It was previously shown in various cortical areas that inhibitory synapses show turnover and that plasticity destabilizes them and causes their loss, and a relationship between size and turnover was also described previously. In addition, there are some major and minor issues that need to be addressed.

Major:
1. Perisomatic inhibitory synapses. The authors find that while gephyrin-GFP puncta on the somata are relatively large, they are surprisingly dynamic and show most turnover. This turnover is not affected by fear condition in contrast to the puncta on the dendrites and AIS. This may be an interesting novel finding. However, gephyrin-GFP is known to form aggregates in the soma if slightly overexpressed. The authors need to show convincingly that the observed puncta are indeed inhibitory synapses, as was shown previously for dendritic puncta (Chen et al). Without this evidence it is well possible that these puncta are actually aggregates. Less worrisome is the analyses of the AIS synapses – but also here I do not believe that it has not been shown previously how well gephyrin-GFP labels GABAergic synapses.

2. In many analyses, statistics are used treating the inhibitory synapses as independent. This is not really justified. It would be better if a nested approach (e.g. linear mixed-effect model) would be used for statistics. At a minimum, the authors need to show that the effects they find are not caused by only a few neurons/dendritic branches/mice

3. I am not sure what insights the modeling of the AIS gephyrin-GFP plasticity/stability really brings. Moreover, it is based on the assumption that "AIS-projecting inhibitory neurons are ideally positioned to control the output of CA1 PNs and indeed they have been shown to control the activity of place cells during spatial navigation." (ref 48: Dudok et al. 2021). The few studies in which the activity of axo-axonic cells (AACs) was altered in vivo, actually do not show this. Dudok et al show that only ~15% of cells were significantly suppressed by AAC activation, and similar results were obtained in frontal cortex (Lu et al Nat. Neurosci. 2017) and visual cortex (Seignette et al 2024). Moreover, the fact that the model does not replicate the increased dendritic inhibitory synapse turnover upon TFC challenges the reliability of the model.

4. Can the authors provide some more data/analyses showing that the CNN approaches that they use for segmentation and quantification approaches are reliable? These seem really useful and powerful approaches and deserve some more attention. Will the code be made available?

Minor:
1. Figure 6b shows that smaller dendritic inhibitory synapses display a trend towards more turnover when they are smaller. Figure 6g shows that inhibitory synapses onto higher order branches show less turnover. Finally, figure 2g shows that inhibitory synapses onto higher order branches are smaller. Have the authors assessed the relationship of inhibitory synapse size and turnover per dendritic order? I wonder whether the relationship may be underestimated due to the larger, but more dynamic inhibitory synapses on the more proximal dendrites. These may be formed by a different interneuron subset than those on more distal dendrites.
2. Page 5: Results "However, it is unclear whether such a relationship holds true over longer durations of time and to what extent INS also show such a relationship." This was actually shown in van Versendaal et al 2012.
3. Page 8: Discussion "Consistent with previous data 42,64,65, also in the live brain we find evidence of a mechanism regulating the size of dendritic spines as well as of INS located on the dendrites and somata of dCA1 PNs by decreasing the size of bigger and increasing the size of smaller synapses at the time scale of days." Is this regulation, or regression to the mean in your analysis? (The largest synapses are simply more likely to become smaller and the smallest ones to become larger).
4. Page 10: Discussion "Turnover of basal dendritic INS increased immediately after TFC and remained high for several days, leading to their destabilization. This is consistent with previous work in the motor cortex 25". This is ref. 27. It is also consistent with inhibitory synapse destabilization in visual cortex upon plasticity induction (refs 25 and 26).
5. Page 13: Methods "Mice were image using either a Bruker Ultima IV microscope - equipped with an InSight Deep See dual color laser system (Spectra-Physics)" image->imaged
6. Please extend the legend of Fig. 8 so it can be understood without having to go back to the results section.

Version 1:

Reviewer comments:

Reviewer #1

(Remarks to the Author)
The authors have responded very adequately to my comments and in my view did so too with respect to the comments and suggestions of the other 2 reviewers. I have no further comments.

(Remarks on code availability)

Reviewer #2

(Remarks to the Author)
The revisions to the manuscript have largely improved the clarity and confidence of the reported findings. The reformatting of the graphs have also made the revised manuscript easier to follow and understand.

Response to major comments
1. Statistical analysis: The authors have comprehensively updated their data analysis approach to account for grouping of measurements within animals/cells. The analysis now incorporates mixed-effects models with nested variables to avoid

treating all data points as independent. Authors provided good justification for use of frequentist statistics (p-values and confidence intervals) for more simple measurements such as size, and Bayesian models in more complex longitudinal measurements such as synaptic survival. The robust analysis approach in the revised manuscripts provides more confidence in the reported results.

2. Excitatory Vs Inhibitory labelling: The authors have removed the direct comparisons between GFP Vs Gephyrin-labelled spines (formerly Fig 5a-b) in acknowledgement of the difference in labelling approaches and have re-phrased discussion to refer to spines rather than excitatory synapses. The authors state they are setting up but not yet practicing equivalent an GFP-Shank labelling approach for excitatory synapses as requested. While not fully addressing the concerns, the revised figure does now show that the difference in putative excitatory and inhibitory "synapse" survival is only evident in newborn and not pre-existing dendrites/INS, which does support the equivalency of this labelling techniques in measuring survival dynamics

3. AIS synaptic clusters: The authors have updated their description of in vivo AIS identification criteria applied and clarified the ex vivo confirmation approach. The limitations of direct comparisons between the stability of single synapses against synaptic clusters (i.e. somatic INS Vs AIS INS) was not resolved, and these comparisons remain as a major component of figure 3. However, the putative influence of synapse clustering on apparent survival was more fully acknowledged in the revised discussion.

4. Discussion: The revised section has been improved with more specific details about proposed contexts e.g. IN population that could explain the result found. Interpretation of data is clearer as a result.

5. Computational modelling: Thank you for clarifying the modelling component.

Response to minor comments
1. AIS identification: Addressed
2. Fig 2e/5a data points: Authors have updated analysis to now look a relative size (ranked) across days (e.g rank order day 1 vs day 2) as shown in figures 2e and 4e. My understanding is that data has been split into subgroups based on how many INS are present on a given neuron. In my opinion, this graph is still a little unclear for example the difference in relative line thickness used to different low vs mid vs high INS containing neurons is very hard to see on the plotted graph. It is also unclear why this subgroup split is included in the plot as I could not find reference to it in the results? Additionally if each line represents a neuron, please include n number somewhere in graph or figure legend.
3. Sampling Frequency: Addressed
4. Fig 3 correlation: Addressed (panel removed)
-Fig 3 data points: Addressed (panel removed)
-Fig 3, panel d: Addressed (panel removed)
-Survival plots: Addressed (Reformatted and mean survival time plot added)
5. Spine Distribution: Addressed
6. Surgical methods: Addressed

New comments
Fig 2d. and 4c What are the empty rows? If an INS isn't present in any of the 13 days (as suggested by empty rows) why is it included?

(Remarks on code availability)

Reviewer #3

(Remarks to the Author)
I co-reviewed this manuscript with one of the reviewers who provided the listed reports. This is part of the Nature Communications initiative to facilitate training in peer review and to provide appropriate recognition for Early Career Researchers who co-review manuscripts.

(Remarks on code availability)

Reviewer #4

(Remarks to the Author)
I co-reviewed this manuscript with one of the reviewers who provided the listed reports. This is part of the Nature Communications initiative to facilitate training in peer review and to provide appropriate recognition for Early Career Researchers who co-review manuscripts.

(Remarks on code availability)

Reviewer #5

(Remarks to the Author)
The authors have addressed most of my concerns with additional experiments and analyses, which I appreciate. However, I remain somewhat unconvinced by the VGAT staining as evidence that somatic Gephyrin-GFP puncta are not aggregates. Colocalization was assessed overall, but not specifically for somatic puncta, and given the high density of VGAT labeling, colocalization may be overestimated. Including a shifted-channel control would help establish chance levels, and this should be feasible with the existing images.

I would therefore encourage the authors to assess colocalization specifically for somatic puncta. If these puncta do represent aggregates, it may be more appropriate to exclude those data to avoid potential misinterpretation. While the current caveat in the results section is helpful, resolving this point more directly would strengthen the manuscript. This is not a reason for rejection, but a strong recommendation.

Finally, "pyramidal neurons" and "PNs" are used throughout the text. Please use "PNs" after introducing the abbreviation.

(Remarks on code availability)

Version 2:

Reviewer comments:

Reviewer #5

(Remarks to the Author)
The authors addressed the last issues I had raised and I have no further comments.

(Remarks on code availability)

Open Access This Peer Review File is licensed under a Creative Commons Attribution 4.0 International License, which permits use, sharing, adaptation, distribution and reproduction in any medium or format, as long as you give appropriate credit to the original author(s) and the source, provide a link to the Creative Commons license, and indicate if changes were made.
In cases where reviewers are anonymous, credit should be given to 'Anonymous Referee' and the source.
The images or other third party material in this Peer Review File are included in the article's Creative Commons license, unless indicated otherwise in a credit line to the material. If material is not included in the article's Creative Commons license and your intended use is not permitted by statutory regulation or exceeds the permitted use, you will need to obtain permission directly from the copyright holder.
To view a copy of this license, visit https://creativecommons.org/licenses/by/4.0/

REVIEWER COMMENTS

Reviewer #1 (Remarks to the Author):

The manuscript by Klimmt et al. examines the structural plasticity of inhibitory synapses (INS) on three main subcellular domains of CA1 principal neurons (PNs): basal dendrites, somata, and axon initial segments (AIS). Using two-photon in vivo imaging, the authors monitor the size and temporal dynamics of these synapses and compare them with spines. They report that dendritic INS are less dynamic than spines during their first acquisitions, but undergo reorganization following trace fear conditioning. They also employ a mathematical model to describe the stabilization mechanisms of AIS INS.

Overall, this is an interesting paper that focuses on the plasticity of inhibitory inputs onto hippocampal PNs. However, there are several concerns, along with a significant lack of detail. Clarification of experimental methods and data analysis is necessary.

We thank Reviewer #1 for recognizing the impact of our work. We also thank Reviewer #1 for taking the time to go through the paper so carefully: as a matter of fact the constructive comments of Reviewer #1 prompted us to completely re-analyze our data and perform additional experiments as well as additional modelling work. This has elevated our paper and we hope to have mitigated their concerns and clarified methods and data analysis to their standards. All changes are in green font for the Reviewer's convenience. Please note that, despite the full re-analysis of the data, the main take home messages of this work are unchanged from the first submission.

Results described in Figure 1

1. The names of AAVs are inconsistent between the figures and the Methods section.

We thank Reviewer #1 for catching that oversight, it is now fixed in the revised version of the manuscript.

2. The results do not emphasize that the analyzed dendritic compartment is limited to basal dendrites. This is only mentioned once in the Introduction and later in the Discussion, where the authors correctly point out previously reported differences between apical and basal dendrites. These differences relate not only to the nature of inputs but also to intrinsic properties (for example: https://doi.org/10.1371/journal.pone.0118482). In my opinion, the results should consistently specify that only basal dendrites were tracked when referring to the dendritic compartment.

We thank Reviewer #1 for pointing out this potential confounding factor, we added the word "basal" in the relevant sections throughout the text (in green) in the revised version of the manuscript.

3. The authors state: "AIS INS were identified by their large size—consistent with cartridge synapses—and confirmed by AnkyrinG immunostaining." The term "large size" should be quantified and expressed in µm.

We thank Reviewer #1 for pointing out this potential confounding factor, we revised that sentence and added the actual measurements to clarify (lines #80-1, in green).

4. Maximum intensity projection (MIP) images are not ideal for identifying precise subcellular localization, particularly in panel c, where the stack is up to 40 µm thick. Given the likely overexpression due to AAV, it would be preferable to show colocalization with the cell membrane, either by rotating the 3D image or by displaying an individual optical slice

where the tomato and GFP signals overlap. This applies to both Figure 1 and Figure S3 (where I assume panel c is also a MIP). The 3D centroid distance plots could also include average distances to the cell membrane.

The limits in resolution of two-photon imaging together with the nature of the labelling do not allow us to obtain the optical detail Reviewer #1 is looking for. More precisely: the tdTomato is cytosolic and not membrane bound and the axial resolution of our two-photon system (with a 0.8NA objective) is around 2µm. This makes the sort of visualization the Reviewer proposes meaningless, as the fact that a green dot overlaps with a red cell (or not) does not necessarily mean that the two structures overlap. Exactly for these reasons, we rely on fusion proteins and sparseness of labelling – rather than optical resolution alone - to obtain labelling specificity. Nevertheless, we revised Figure S1 to show examples of optical slices depicting dendritic, somatic and AIS INS.

In the same vein, staining for presynaptic markers (e.g., Neuroligin 2 or VGAT) would support the claim that the observed structures are indeed synapses. Without such evidence, the term "putative synapses" would be more appropriate.

While, this approach has been already validated by electron microscopy (see reference 25. *Chen, J.L., Villa, K.L., Cha, J.W., So, P.T.C., Kubota, Y., and Nedivi, E. (2010). Clustered Dynamics of Inhibitory Synapses and Dendritic Spines in the Adult Neocortex. Neuron 74, 361–373.* and reference 27. *Versendaal, D. van, Rajendran, R., Saiepour, M.H., Klooster, J., Smit-Rigter, L., Sommeijer, J.-P., Zeeuw, C.I.D., Hofer, S.B., Heimel, J.A., and Levelt, C.N. (2012). Elimination of Inhibitory Synapses Is a Major Component of Adult Ocular Dominance Plasticity. Neuron 74, 374–383),* the Reviewer's concern is important. Thus we performed the experiment suggested by Reviewer #1. To this aim, we infected a new groups of animals (as described in the manuscript) and performed immunostaining for the protein vGAT (that specifically localizes on inhibitory presynapses). Out of 466 Gephyrin-GFP puncta examined by confocal microscopy, 91% colocalized with vGAT immunofluorescence, thus demonstrating that the vast majority of Gephyrin-GFP puncta are indeed in close proximity (within the limits of confocal optical microscopy resolution) with an independently identified INS. We hope this will convince Reviewer #1 of the validity of our labelling approach. We revised Fig S1 to add these new results (panels a to c).

The same concern applies to Figure 2. Figure 4 does not specify whether the images are stacks or single slices.

Panels a and b in Figure 2 were obtained from the same dataset as Figure 1, thus we refer Reviewer #1 to our answer to their previous comment. We apologize for the oversight in the legend of Fig 4, we have now added the relevant information (lines #1042-4, in green for the Reviewer's convenience).

Behavioral protocols and results raise the most significant concerns:
1. You used a cued fear memory test and investigated structural plasticity in CA1. However, the amygdala is more strongly implicated in this type of memory (e.g., https://doi.org/10.1186/s13041-020-00652-y and https://doi.org/10.1037//0735-7044.106.2.274).

We used a Trace Fear Conditioning (TFC) paradigm (as specified in the Abstract, Introduction, Results and Methods sections). In this paradigm there is a delay of 20 s between the end of the tone and the delivery of the shock. The dorsal hippocampus has been shown to play a key role in this associative paradigm. In fact, converging lesion, pharmacological, and optogenetic studies show that inhibiting the dorsal hippocampus, particularly dorsal CA1, impairs learning in trace fear conditioning in mice. Inactivation of NMDA receptors by APV local perfusion in the dorsal hippocampus reduce freezing to the tone after trace fear conditioning, indicating that normal dorsal hippocampal activity is required to acquire the trace association (*Misane I, Tovote P, Meyer M, Spiess J, Ogren SO, Stiedl O. Time-dependent involvement of the dorsal hippocampus in trace fear conditioning in mice. Hippocampus. 2005;15(4):418-26*). Lesions of the dorsal hippocampus also attenuate auditory trace fear memory (*Bangasser DA, Waxler DE, Santollo J, Shors TJ. Trace conditioning and the hippocampus: the importance of contiguity. J Neurosci. 2006 Aug 23;26(34):8702-6*). Optogenetic silencing of dorsal CA1 pyramidal neurons during trace fear training lowers subsequent freezing to the cue (*Wilmot JH, Puhger K, Wiltgen BJ. Acute Disruption of the Dorsal Hippocampus Impairs the Encoding and Retrieval of Trace Fear Memories. Front Behav Neurosci. 2019 May 29;13:116*). Even brief inhibition of dorsal CA1 for tens of seconds immediately after each shock early in training is sufficient to reduce later trace fear (*Kyle Puhger, Ana P. Crestani, Cassiano R.A. F. Diniz, Brian J. Wiltgen, The hippocampus contributes to retroactive stimulus associations during trace fear conditioning, iScience, Volume 27, Issue 3, 2024*). The papers Reviewer #1 cites refer to cued FC where the tone and the shock overlap.

2. In Figure 7b, you compare Habituation A with Recall A, and Habituation B with Recall B. I assume Habituation B refers to the time in B before the tone, although this is never clearly stated.

The assumption of Reviewer #1 is correct, we have revised the Methods section to increase clarity (lines #418-9, in green for the Reviewer's convenience).

How about comparing Recall A and Recall B? Significantly lower freezing in Recall B could suggest contextual learning, thus supporting your analysis, conclusions, and the article's title (here, the word basal should be added before dendrite). On the other hand, similar levels of freezing in both conditions would suggest an absence of learning, which would contradict the title. This cannot be assessed from the figure alone, and detailed data (e.g., averages and S.E.M. values) are not reported.

With the TFC paradigm we applied here, contextual learning is assessed by measuring freezing during Recall A in Context A. Context B is a novel context for the mice and introduced on the day of Recall. Only the tone memory associated to the shock is probed in this context as Context B is not associated with a shock. Therefore, it is unclear to us, what insights in contextual learning the Reviewer would gain by comparing the freezing in Recall A and B. Nevertheless, we tested it statistically and animals froze to the same levels (non-significantly different according to pairwise Wilcoxon non parametric repeated measurement test, $p = 0.14$) to context (Recall A) and to tone (Recall B). The fact that the animals significantly freeze in the context, without tone (Recall A), means they learned the association between the context and the shock, while the fact they freeze to the tone, in a different context (Recall B), means they learned the association between the tone and

the shock. So our data show both contextual and tone associations. It is unclear to us how *"similar levels of freezing in both conditions would suggest an absence of learning",* in fact, our data rather shows that animals learn two different aspects of the task.

3. Additionally, the time lapse ends just one day after the recall. The remote recall phase is generally the one more dependent on hippocampal activity.

I am afraid also here we do not understand the relevance of the Reviewer's comment. While we of course agree that the hippocampus is important during recall as well as it is during encoding, we fail to understand how does this impact the significant effects we detect after fear learning. We agree it would be interesting to study the effect of recall but this is outside the scope of this work that overtly focuses on encoding. In addition, the effects we detect in our work are in line with literature showing changes in synaptic densities in CA1 already within 1-24h post recall (*Restivo, L., Vetere, G., Bontempi, B., & Ammassari-Teule, M. (2009). The formation of recent and remote memory is associated with time-dependent formation of dendritic spines in the hippocampus and anterior cingulate cortex. Journal of Neuroscience, 29(25), 8206-8214.)*

4. The inputs relevant fear context memory—if learning did occur—would primarily target apical dendrites (from the medial entorhinal cortex), and to a lesser extent, basal dendrites.

The role of basal dendrites of the dorsal CA1 – which receive direct CA3 input – to fear memory has been demonstrated. In fact, contextual fear learning selectively strengthens a subset of CA3–CA1 synapses located on basal dendrites and engages CA1 dendritic "engram" spines that are preferentially connected with CA3 engram cells (*Lee C, Lee BH, Jung H, Lee C, Sung Y, Kim H, Kim J, Shim JY, Kim JI, Choi DI, Park HY, Kaang BK. Hippocampal engram networks for fear memory recruit new synapses and modify pre-existing synapses in vivo .Curr Biol. 2023 Feb 6;33(3):507-516).* In addition, our group previously found a correlation between intrinsic dynamics of dendritic spines located on basal dendrites of the dCA1 and contextual fear recall (*Castello-Waldow TP, Weston G, Ulivi AF, Chenani A. Loewenstein Y. Chen A and Attardo A***.** *Hippocampal neurons with stable excitatory connectivity become part of neuronal representations. PLoS Biol 18(11): e3000928*). Thus, while involvement of dCA1 basal dendrites might be lower than the one of apical dendrites in fear learning, it is evident. Hence it should not come as a surprise that we find learning-related effects on synapses located on basal dendrites.

Incidentally, projection-specific manipulations showed that dCA3 to dCA1 projections are necessary for precise contextual fear discrimination, as inhibition of this pathway impaired discrimination between similar contexts (*Besnard A, Miller SM, Sahay A. Distinct Dorsal and Ventral Hippocampal CA3 Outputs Govern Contextual Fear Discrimination. Cell Rep. 2020 Feb 18;30(7):2360-2373.e5*). Thus showing the involvement of the dCA3 in learning a fear association.

5. Analyses concerning gain, loss and turnover of INs and spines (Figures 7c,f,i,l and S7) should compare TFC mice with control mice of Figure 3d,f. Comparing 4 days before and after training as done in Figure 7 d,g,j,m is informative but

not exactly the same. The simple fact of placing mice in a different environment, under the microscope, may trigger structural plasticity mechanisms even without TFC experience.

Here the Reviewer raises two interesting points.

First, the Reviewer argues that a between group comparison is a better way to analyze the data than a within group comparison. We disagree with this statement as the between group comparison is negatively affected by the two different and independent noise sources in the two groups: individual mice differences and different experimental batches, of which the first one is the most prominent as different mice are also sampled in a different way. The within group comparison, on the contrary, is not affected by the individual variability of mice, thus it has an higher statistical power.

Second, the Reviewer points out that imaging itself might lead to changes in plasticity. This is valid point, which we took very seriously. For this reason we repeated the analyses of turnover for Figure 7 taking into account the temporal structure of the Control group. In our new analysis, the non-TFC control group serves as the primary null reference to distinguish specific TFC-induced effects from common ongoing background drift in turnover stemming from the experiments' longitudinal imaging design. Thus, for inference we applied a hybrid criterion to identify robust learning-induced plasticity. Significant effects were defined as time-points satisfying two conditions: (i) the Experimental group exhibited a significant deviation from its own baseline (assessed via a Probability of Direction > 0.95), and (ii) there was substantial Bayesian evidence for a *Group* x *Phase* interaction (Probability of Direction > 0.95), confirming that a potential TFC induced deviation in the experimental group was distinct from drift-caused deviations in the control group. These are the significances reported in Figure 7 d, g, j of the revised manuscript. We thank the Reviewer for this insightful comment and we hope this takes care of their concern.

Statistical concerns:
The authors should specify the statistical tests used for each subfigure and provide complete statistical information.
In most analyses, INS are used as the unit of analysis (n), pooling data across different time points, neurons, and animals. However, the number of animals is only reported for the behavioral experiment and in Figures S1, S4, and S6. The total number of animals is not mentioned in the Methods section.

We thank the Reviewer for pointing this out, we added the number of animals in the Methods and improved throughout the data presentation, including more raw data and information about neurons and mice when applicable also in the main figures and legends.

In the trace fear conditioning experiments, synapse-related data appear to disregard individual animals—or at least this is not clearly stated. Ideally, a mixed-effects model (as described in Figure 5) should be applied across all statistical analyses (e.g., https://doi.org/10.1016/j.celrep.2019.05.098). This would allow the use of larger n samples for comparisons (i.e. synapses) taking into account the variability from different animals.

We thank the Reviewer for pointing this out, we took on this advice wholeheartedly and reached out to an expert in statistical modelling – the second joint first author of the revised version of the manuscript – and re-analyzed our data with mixed-effects models as the Reviewer suggested.

Because synapses are nested within dendrites, neurons and mice, we implemented mixed-effects models with nested random intercepts for mice, neurons and where appropriate dendrite ID across all inference analysis throughout the paper to account for potential non-independence in the data. For the TFC experiment, we additionally included mouse and neuron-level random slopes allowing interval-specific deviations of individual animals, accommodating inter-animal variability in the temporal response to TFC yielding rigorous and conservative inference on population level fixed effects. Furthermore the control data (non TFC-cohort) was additionally included into the TFC inference to distinguish true TFC-induced population effects from baseline drift.

Figure 6d should present dot plots.

We understand this comment as the request of showing a more detailed version of the data. Thus figure 6 now shows scatter plots detailing three levels: (i) the raw values per synapse (dots), (ii) the means per neuron (circles) and (iii) the mice (colors).

When the authors claim that somatic INS turnover was higher than in dendritic or AIS compartments, they assert that this was not due to positional fluctuations. It would be helpful to clarify how this was demonstrated.

As described in the paragraph “Long-term dynamics of INS are compartmentalized on dCA1 PNs” and Figure S3, to investigate the positional stability of INS we compared the temporal variability of the position of INS to the temporal variability of the position of a series landmark that are expected to be stable through time (such a bifurcation points of dendrites). To this aim we wanted to be sure to track stable INS, thus we imaged 150 INS over time as quickly as our technique allowed us (every 10 minutes) and calculated the positional fluctuation of each of the items we tracked over time. As we did not find any significant difference between the temporal variability of INS and of landmarks that are supposed to be stable we concluded that also INS were stable and any residual variability to be due to tissue motion and measurement noise. We have revised the relevant text (Lines #120-8, in green for the Reviewer’s convenience) to increase clarity.

Minor issues in the Discussion:
1. In the paragraph “Subcellular compartmentalization of INS dynamics,” the authors mention redundancy of somatic INS compared to dendritic ones, possibly reflecting a smaller individual effect. Were multiple somatic INS significantly more abundant than dendritic INS in this study? The reported numbers suggest otherwise.

We believe Reviewer #1 refers to the sentence: *“while somatic-projecting inhibitory neurons have multiple INS on the soma of the same postsynaptic PN”*. This statement is backed by literature as we do not image the axons of the inhibitory presynaptic neurons. The reported numbers of sampled synapses per compartment are also dependent on the field of view imaged which individually limits the number of synapses per compartment we can sample per neuron and does not reflect the overall total numbers of synapses per compartment. Hence we cannot make a quantitative statement about this but only a qualitative one.

2. In "Dendritic INS are less dynamic than spines," the phrase "Notably, the inhibitory connectivity between the basal and the apical domain…" is unclear. Did the authors mean onto both domains, rather than between them?

In the revised version this sentence has been removed.

3. In "The relationship between size and persistency in dendritic spines and INS," the sentence "Size decrease 48h before disappearance…" contains the word slated—this appears to be a typo and should likely be selected.

In the revised version this sentence has been removed.

Reviewer #2 (Remarks to the Author):
The manuscript by Klimmt et al monitors structural plasticity of inhibitory synapses impinging onto pyramidal neurons in dorsal CA1 using longitudinal two-photon imaging. The authors test the hypothesis that structural plasticity is specific to sub-cellular compartments: somatic, initial axon segment and dendritic. In addition to passive recordings, the authors study the effect of hippocampal-dependent learning on synaptic changes and recapitulate the compartmentalized difference in a computational model. Structural inhibitory synapse plasticity has been studied in the cortex by a number of groups but plasticity in hippocampus has mostly been studied by one group in a series of papers detailing structural inhibitory plasticity during learning (PMID: 26807952, PMID: 24336286, PMID: 25695271). The authors report differences in stability of inhibitory synapses between compartments and also compared to excitatory synapses with compartment specific effects claimed as a result of learning. The results are therefore interesting but there are serious problems with the analysis and statistical approaches that significantly reduce confidence in the conclusions.

We thank Reviewer #2 for the interest in our results. To fully address their concerns we have completely re-analyzed all data in the revised version of the manuscript (see answers to specific comments). We are pleased to confirm that the main conclusions the Reviewer found interesting in the first version of the manuscript, stay unchanged in the revised version. We also thank the Reviewer for pointing out the excellent work of the Caroni group, of which we are of course aware and that is now cited in the revised version of the manuscript.

Major Comments
1. For statistical analysis, authors used grouped comparisons that treated individual synapses as independent data points regardless of if they were obtained from the same neuron. This would impact the variance between the somatic, AIS, dendritic defined synapses as many more dendritic INS (≤85) relative to somatic (≤20) and AIS (≤16) INS may be sampled from within the same neuron. As the activity history of an individual neuron during its lifetime would likely make these INS more similar to each other than to INS from another distinct neuron these should be accounted for. This becomes even more problematic in the longitudinal imaging studies in which an individual INS may be counted as several independent data points if present/persistent across multiple sessions which will directly influence effect size observed. Data needs to be reanalysed with mixed models that include mouse and/or neuron ID as random variables to account for this. The same issues are relevant to the analysis of spines and excitatory synapses.

We thank Reviewer #2 for their insightful comment. We have taken this comment very seriously and reached out to an expert in statistical modelling – who became the joint first author of the revised version of the manuscript –. Data throughout the manuscript have been reanalyzed using mixed-effects models with nested random intercepts at the mouse, neuron, and where appropriate compartment and dendrite levels, directly accounting for the non-independence of synapses sampled from the same neuron and neurons sampled from the same animal. This applies uniformly to all analyses of dendritic INS, somatic INS, AIS INS, spines,

and excitatory synapses. Temporal dependency was also accounted for by either doing day-wise inferences or accounting for the longitudinal structure of the data directly in the models. For longitudinal analysis of TFC effects, random slopes were included at the mouse and neuron level, allowing each biological unit its own temporal trajectory and ensuring population-level TFC effects reflect genuine manipulation-driven signal rather than being biased by between-unit heterogeneity. Models additionally account for overdispersion and potential heteroscedasticity where necessary. Additionally, for the TFC experiment, the non-TFC control cohort was included in all models to formally distinguish TFC-induced effects from baseline temporal drift. This is detailed in several different places in the revised version of the manuscript, and in detail in the methods (in green for the Reviewer's convenience).

2. Comparisons between putative excitatory (dendritic) and inhibitory (gephyrin) synapses are made and several conclusions drawn about differences in their overall size kinetics, however the synapses were labelled using very different methods. Dendritic labelling was measured through cell wide GFP expression in Thy-GFP mice, in comparison with more punctate inhibitory synapse labelling achieved through sparse viral labelling of inhibitory scaffolding protein expression. This introduces a large potential difference size and dynamic measurements which is not appropriately addressed. To confirm that the dynamic differences reported between excitatory synapses and INS are real and not an artifact of experimental design would need to be replicated using a similar labelling approach to GFP-Gephyrin for the excitatory synapses eg GFP-Shank.

We agree with Reviewer #2 that the direct size comparisons between dendritic spines and dendritic Gephyrin puncta are problematic, even only because of the comparison of volumes to areas, respectively. For this reasons we decided to remove these comparisons. However, we do stand by our direct comparison between temporal dynamics of dendritic spines and dendritic INS. In fact, the dynamics of dendritic spines are used as proxies for dynamics of excitatory synapses. In the revised version of the paper we stressed this point by stating it more strongly in the discussion section (Paragraph "Newborn dendritic INS are less stable than newborn spines", line 283) and by referring to dendritic spines – rather than to excitatory synapses – throughout.

Performing direct labelling of excitatory synapses – by PSD95-GFP or GFP-Shank –, is something we are currently setting up but whose fruition is still relatively far in the future. Thus, while we recognize the importance of the experiment, we believe it is outside of the scope of this work which mainly focuses on the effect of learning on INS. We hope that Reviewer #2 will agree with this.

3. The authors concede that it is not possible to distinguish individual inhibitory synapses from clusters using their imaging approach and they therefore treat clusters as individual synapses. This confounds many of the conclusions. For example, the claim is that AIS synapses are larger than other compartments but this could simply be because AIS synapse clusters contain greater numbers of synapses – which is documented. Then AIS clusters of synapses will be more stable simply because there are more synapses there. There is further circularity in the definition of AIS synapses since they are identified by having a large size.

Throughout the manuscript we have been always very candid about the limitation of the optical resolution of our technique. In fact, regarding "*the claim is that AIS synapses are larger than other compartments*" we openly stated – almost verbatim – that "*this could simply be because AIS synapse clusters contain greater numbers of synapse*" twice: in the Results section (page 3, end of first paragraph of "Dynamics of INS are compartmentalized on

dCA1 PNs") and in the Discussion section (page 8, end of first paragraph of "Subcellular compartmentalization of INS size"). These statements are unchanged in the revised version of the manuscript.

We fully agree that "*AIS clusters of synapses will be more stable simply because there are more synapses there",* and we thank the Reviewer for the comment that gave us to the chance to point this out in the revised version of the manuscript (Line 278-82, in green for the Reviewer's convenience).

Finally, regarding the "*circularity in the definition of AIS synapses since they are identified by having a large size."*. We apologize for the lack of clarity: AIS INS were identified *in vivo* by being located on the putative AIS – that is an arborization originating on the basal aspect of the soma and being visibly thinner than dendritic bases – and distinguished by dendritic INS by their larger size. AIS INS (as labelled *in vivo*) overlapped with AnkyrinG immunostaining in *ex vivo* preparations. We have revised the Results section (Lines 80-3, in green for the Reviewer's convenience), to increase clarity.

4. The interpretations given within the discussion section are quite superficial and don't propose many circuit or micro-circuit level connections to explain the results found. For example: "compartmentalization might stem from the specific activity patterns or genetic makeup of pre-synaptic inhibitory neurons" what kind of activity patterns in what types of inhibitory interneurons could explain the observation? "inhibitory connectivity between the basal and apical domain is very different" - how are they different? It would be better to back up the hypothesis with some specific examples.

We have updated the discussion section to include more detailed examples as requested by the Reviewer (Lines 249-52), and we have re-written the discussion regarding comparative stability of dendritic INS and spines (Lines 283-91).

5. The modelling of inhibitory connectivity in Fig8 assumes changes in AIS INS whereas it is well documented that somatic and dendritic plasticity also occurs. The modelling therefore feels preliminary and simply confirms the experimental findings. To add real value the model could be used to investigate the effect of all the observed INS plasticity.

I am afraid we don't fully understand this comment from the Reviewer. Our model assumes changes in all subcellular compartments and it is tuned to reproduce all the dynamics we detect during the control experiment, definitely in AIS INS but also in dendritic and somatic INS. The model does indeed confirm the experimental findings regarding TFC and regarding the difference in survival between pre-existing and new synapses, this shows the consistency of the model. In addition, - when we probe the function of the AIS-projecting inhibitory neurons - the model provides insights into the function of these neurons. We believe this is very real value.

We fully agree that it would be interesting "*to investigate the effect of all the observed INS plasticity"* and, as a matter of fact, we have an ongoing collaboration with Prof. Kappel (second author of the manuscript) to explore this. However, we strongly feel that this upcoming work should be part of a second – fully computational – paper and adding it to the current manuscript would dilute the current focus. We hope that the Reviewer will agree with us.

Minor comments

1. More information required on the identification and classification of AIS INS. The manuscript states "identified by large size" but requires more detail. Was the ankyrin G confirmation conducted in the subset of cases or for all AIS?

Given that being able to identify INS *ex vivo* after *in vivo* imaging is exceedingly difficult, we carried out the Gephyrin validation in an independent subset of animals in which INS were labelled as in the *in vivo* imaging experiments. We clarified this in the Results section (Lines 82-4, in green for the Reviewer's convenience).

2. In Fig2 what do individual data point represent in panel e and similar for Fig 5a? Not stated and not clear.

Following re-analysis of the data, we decided to use a different measure of similarity across time (revised Figures 2e and 4e), hence these panels and their description have been eliminated.

3. What was the sampling frequency? It is stated as "as often as our technique allowed in a new set of mice" but please provide actual sampling frequency.

We believe this question refers to Figure S3. The imaging frequency is 10'. This is reported in revised Figure S3c as numbers in the image panels, and in S3d, e as ticks on the horizontal time line. To satisfy the Reviewer's request we have also added this info to the revised Results section (Line 120-8, in green for the Reviewer's convenience).

4. In Fig3 what does "correlation between gain and loss was higher for somatic than for dendritic and AIS INS" mean biologically? That the overall number of INS remained consistent despite higher rates of loss and gain? This is not clear.

Originally, in panel 3d we meant to indicate that a higher correlation between gain and loss at the level of single neurons (such as the one seen in somatic INS) implies that in each neuron gain and loss are very tightly linked (with even a slight change in the one being immediately balanced by a corresponding change in the other). Conversely, a lower correlation (such as the one seen in dendritic INS) implies that neurons don't always compensate gain and loss in dendritic INS thus some neurons might gain more than they loose on one day and *vice versa* on another day. However, after careful consideration of the statistics of this comparison and of the focus of the paper, we decided to remove these statements and the corresponding figure.

In addition, why does only somatic INS have multiple data points when legend states that each time point is represented?

Only somatic INS had more than one significant value of Spearmann correlation (panel 3c) . However, after careful consideration of the statistics of this comparison and of the focus of the paper, we decided to remove this panel.

And how are the correlations between somatic and AIS compared statistically as there is also a sig positive correlation between loss and gain in AIS shown in panel d?

Indeed, in consideration of the statistics of this comparison and of the focus of the paper, we decided to remove this panel.

It might be easier to visualise if plotted as the average lifespan of INS present at day 1 and new INS present from day 3 rather than as survival fractions.

We thank the Reviewer for this comment. Indeed, in the revised version of the manuscript all panels showing survival of INS or dendritic spines (Fig 3d-e, Fig. 4i and figure 5a, b) also report the population-level Restricted Mean Survival Times per group and used this for statistical comparisons.

5. In referring to Fig4 the statement "Distribution of dendritic spines' volumes was skewed towards higher values" is unclear. There is no statistical comparison here. Does this mean higher volumes than dendritic INS or a non-gaussian distribution of size within the population.

Apologies for the confusion, this statement refers to the non-gaussianity of the distribution. We have updated the revised version to clarify this (Lines 96 and 134-5, in green for the Reviewer's convenience).

6. In the methods much more information about surgical procedures is required including anaesthesia, analgesia, window size, aspiration fluid etc.

We have added this information in the revised version of the manuscript (Lines 401-9, in green for the Reviewer's convenience). The cited JoVE paper (Ulivi *et al.,* 2019) includes a detailed video description of the hippocampal implantation filmed in our lab as well as a complete list of all materials in the supplement.

Reviewer #3 (Remarks to the Author):
I co-reviewed this manuscript with one of the reviewers who provided the listed reports. This is part of the Nature Communications initiative to facilitate training in peer review and to provide appropriate recognition for Early Career Researchers who co-review manuscripts.

Reviewer #4 (Remarks to the Author):
I co-reviewed this manuscript with one of the reviewers who provided the listed reports. This is part of the Nature Communications initiative to facilitate training in peer review and to provide appropriate recognition for Early Career Researchers who co-review manuscripts.

Reviewer #5 (Remarks to the Author):
In this study, the authors analyzed the structural plasticity of inhibitory and excitatory synapses onto pyramidal cells in the CA1 area of hippocampus over a period of two weeks in control mice and in mice undergoing trace fear conditioning

(TFC). To image inhibitory synapses, sparse pyramidal neurons were labeled with dtTomato to visualize the structure of the neurons and gephyrin-GFP to label inhibitory synapses. For spine imaging, Thy1-GFP transgenic mice were used.. Chronic two-photon microscopy was employed to repeatedly image the same neuronal structures, allowing the quantification of synapse numbers and sizes, and their dynamics. Inhibitory synapses at dendritic branches, somata and axon initial segments (AIS) were imaged, as well as dendritic spines in a separate experiment.
The major observations are that:

- *-Inhibitory synapses at dendritic branches show turnover, but less so than inhibitory synapses onto the AIS. Somatic gephyrin-GFP puncta are the least stable.*
- *-Dendritic inhibitory synapses are smaller than those on somata or AIS (though this is probably a compound labeling of multiple AIS synapses).*
- *-Dendritic spines are more dynamic than inhibitory synapses on dendrites, but less so than gephyrin puncta at the soma.*
- *-Inhibitory synapses at higher order dendrites are more stable.*
- *-Dendritic spines, AIS inhibitory synapses and dendritic inhibitory synapses show a relationship between size and persistence, with smaller synapses being less persistent (though not significantly so for dendritic inhibitory synapses).*
- *-Fear conditioning destabilizes inhibitory synapses on dendrites while it stabilizes those on the AIS. It (surprisingly) does not affect turnover of dendritic spines or somatic inhibitory synapses.*
- *-Finally, the authors use a modeling approach to study what factors might influence AIS inhibitory synapse stability, and find that this includes the proportion of AIS targeting cells, making the AIS synapses excitatory instead of inhibitory, the influence of disinhibitory neurons and the sign of the effect (inhibitory/excitatory) of AIS synapses.*

This study was a tour-de-force. The authors used challenging approaches to analyze inhibitory synapse turnover over several weeks, and developed novel tools to segment and analyze the synaptic structures. This work represents an impressive amount of work that was carried out thoroughly.
At the same time, while the findings are novel, they mostly align with what has previously been shown in neocortical areas. It was previously shown in various cortical areas that inhibitory synapses show turnover and that plasticity destabilizes them and causes their loss, and a relationship between size and turnover was also described previously. In addition, there are some major and minor issues that need to be addressed.

We thank Reviewer #5 for recognizing the scope and the technical depth of our work. While it is true that our results substantially agree with the neocortical literature, we are the first ones to show a coordinated (and diverging in sign) effect on dynamics of INS upon learning. We believe that conceptual advance linked to this finding makes our work stand out. We hope to be able to satisfy all of the Reviewers' concerns in the revised version of the manuscript and in our point-by-point detailed answer.

Major:
1. Perisomatic inhibitory synapses. The authors find that while gephyrin-GFP puncta on the somata are relatively large, they are surprisingly dynamic and show most turnover. This turnover is not affected by fear condition in contrast to the puncta on the dendrites and AIS. This may be an interesting novel finding. However, gephyrin-GFP is known to form aggregates in the soma if slightly overexpressed. The authors need to show convincingly that the observed puncta are indeed inhibitory synapses, as was shown previously for dendritic puncta (Chen et al). Without this evidence it is well possible that these puncta are actually aggregates. Less worrisome is the analyses of the AIS synapses – but also here I do not believe that it has not been shown previously how well gephyrin-GFP labels GABAergic synapses.

The Reviewer's concern is important and to answer it we decided to test whether the viral Gephyrin GFP puncta would colocalize with the protein vGAT, that specifically localizes on inhibitory pre-synapses. Thus we infected a new groups of animals (as decribed in the manuscript) and performed immunostaining for the protein vGAT. Out of 466 Gephyrin-GFP puncta located in all PNs subcellular compartments we examined by confocal microscopy, 91% colocalized with vGAT immunofluorescence, thus demonstrating that the vast majority of Gephyrin-GFP puncta are indeed in close proximity (within the limits of confocal optical

microscopy resolution) with an independently identified INS. We revised Fig S1 to add these new results (panels a to c). Nevertheless. as we cannot fully exclude that at least some of the somatic Gephyrin-GFP puncta we imaged are actually aggregates due to overexpression and not INS, we added a sentence to the relevant section in the discussion (lines 262-4, in green for the Reviewer's convenience). In addition, for completeness, we also added a paragraph to specify how clustering might affect apparent stability of AIS INS (lines 278-82, in green for the Reviewer's convenience).

2. In many analyses, statistics are used treating the inhibitory synapses as independent. This is not really justified. It would be better if a nested approach (e.g. linear mixed-effect model) would be used for statistics. At a minimum, the authors need to show that the effects they find are not caused by only a few neurons/dendritic branches/mice.

We thank the Reviewer for pointing this out, we took on their advice very seriously and reached out to an expert in statistical modelling – the second joint first author of the revised version of the manuscript. In the revised manuscript we explicitly address dependence in our data using hierarchical mixed-effects models throughout our statistical analyses. All models included random intercepts for mouse and neuron identity (and, where appropriate, dendritic branch or compartment), thereby accounting for the nesting of synapses within neurons and animals and preventing synapses from being treated as independent observations. For analyses testing TFC-related changes, we additionally included random slopes on the mouse:compartment and mouse:neuron:compartment levels allowing TFC effects to vary across mice and neurons. Together, these measures substantially reduce the risk that results are driven by a small number of units with particularly strong responses by accounting for inter-animal and inter-neuron variance to estimate the population-level fixed effects. Additionally, for the TFC experiment, the non-TFC control cohort was included in to formally distinguish TFC-induced effects from baseline temporal drift. To further increase transparency we also included plots of the raw data for all major analyses in the revised manuscript grouped by mouse and/or neurons.
This is detailed in green throughout the text for the Reviewer's convenience. Please note that, despite the full re-analysis of the data, the main take home messages of this work are unchanged from the first submission.

3. I am not sure what insights the modeling of the AIS gephyrin-GFP plasticity/stability really brings.

This is – to the best of our knowledge – the first attempt to investigate the link between the function of a class of inhibitory neurons specified by their synaptic connectivity patterns and the structural dynamics of INS in the dCA1 microcircuitry. The model uniquely enabled us to probe changes in the circuitry and specific wiring of the interneurons that cannot not be biologically tested with currently available tools.

Moreover, it is based on the assumption that "AIS-projecting inhibitory neurons are ideally positioned to control the output of CA1 PNs and indeed they have been shown to control the activity of place cells during spatial navigation." (ref 48: Dudok et al. 2021). The few studies in which the activity of axo-axonic cells (AACs) was altered in vivo, actually do

not show this. Dudok et al show that only ~15% of cells were significantly suppressed by AAC activation, and similar results were obtained in frontal cortex (Lu et al Nat. Neurosci. 2017) and visual cortex (Seignette et al 2024).

We thank Reviewer #3 for pointing this out, we have rephrased the result section to reflect the Reviewer's comment (lines 197-9, in green for the Reviewer's convenience). In fact, regarding whether AAC control over PNs in CA1 is inhibitory, we could test with the model, how the dynamics after learning changed when we artificially reversed the sign of AAC action to be positive (Fig. 8 o, p, q). Under these conditions we found that the stabilization of AIS INS post learning was impaired. This exemplifies an experiment that would be impossible to test biologically and makes the model a valuable tool to infer mechanisms that could underly the effect we observe in our data.

Moreover, the fact that the model does not replicate the increased dendritic inhibitory synapse turnover upon TFC challenges the reliability of the model.

We thank Reviewer #3 for pointing this out, this prompted us to re-evaluate our modeling approach. As a matter of fact we realized that the baseline and conditioned contexts in our simulations were unreasonably similar. We thus run again the simulated TFC experiment and simply increased the differences between the baseline and conditioned context (as described in the methods section lines 686-94 and in the revised figure 8,panel b) while leaving all other model parameters untouched. This led to a better fit between the modelled INS turnover and the experimental one. This is now shown in the revised figure 8 (panels d and e) where the model was able to reproduce both the instability of new INS as well as the effects of TFC on INS we detected *in vivo*.

4. Can the authors provide some more data/analyses showing that the CNN approaches that they use for segmentation and quantification approaches are reliable? These seem really useful and powerful approaches and deserve some more attention. Will the code be made available?

The CNN-based segmentation and volumetric analysis we used here is derived from the *SpineS* framework (Argunşah et al., *Scientific Reports*, 2022). In *SpineS* the models were validated against expert manual annotations and demonstrated robust performance across diverse imaging conditions. Since its publication, the same CNN architecture has been reliably applied in several studies investigating structural plasticity, including work on stimulation-dependent long-term spine dynamics (Argunşah & Israely, *iScience*, 2023), heterosynaptic structural changes (Argunşah & Israely, *Frontiers in Cellular Neuroscience*, 2023), receptor-dependent alterations in ASD models (Perera-Murcia et al., *J. Neuroscience*, 2025), Fragile X–related synaptic dysfunction (Ramiro-Cortés et al., 2026), and homeostatic scaling of spine size (Hobbiss et al., *iScience*, 2018). The consistent use of this pipeline across laboratories and experimental paradigms highlights its reliability and generalizability. The original *SpineS* code, including the CNN models used for segmentation and spine-volume estimation, is fully open source. An updated version retrained specifically for *in vivo* time-series imaging will also be made publicly available upon publication.

Minor:
1. Figure 6b shows that smaller dendritic inhibitory synapses display a trend towards more turnover when they are smaller. Figure 6g shows that inhibitory synapses onto higher order branches show less turnover. Finally, figure 2g shows that inhibitory synapses onto higher order branches are smaller. Have the authors assessed the relationship of inhibitory synapse size and turnover per dendritic order?

This is a very interesting question to address and we extended the analysis to answer it. To directly test the effect of dendritic order and size on the stability of dendritic spines and INS independently we modelled the interaction between dendritic order and synapse size using a Bayesian accelerated failure time (AFT) model. We computed the predicted restricted mean survival time (RMST) after standardizing size to a single typical value across all orders isolating order effects independent of size differences. Additionally we compared survival between synapses at the 25th and 75th size percentile separately within each dendritic order, as well as marginalized across all orders. We found in both dendritic spines as well as dendritic INS significant isolated effects of dendritic order on RMST, while significant size effects on RMST were only found in dendritic spines.

I wonder whether the relationship may be underestimated due to the larger, but more dynamic inhibitory synapses on the more proximal dendrites. These may be formed by a different interneuron subset than those on more distal dendrites.

This is a very concrete possibility. In fact, many different interneuron subtypes contact the basal dendritic compartment, e.g. Bistratified Cells (PV+, SOM+), Basket Cells (PV+ or CCK+ or both) and most abundant are Ivy Cells (NPY+). Some of these cells also contact the soma. All these connections are differentially influenced by relative abundance of interneurons but also on how these interneurons distribute their synapses on the pyramidal neuron (see Bloss *et al.*, 2016). There is no clear-cut answer on which interneuron type drives the differences we observe since the synaptic population is heterogenous. We added more detailed considerations in the discussion. (Lines 263-9 and 313-7).

2. Page 5: Results "However, it is unclear whether such a relationship holds true over longer durations of time and to what extent INS also show such a relationship." This was actually shown in van Versendaal et al 2012.

Thank you for catching that missing information, we rephrased the Results section.

3. Page 8: Discussion "Consistent with previous data 42,64,65, also in the live brain we find evidence of a mechanism regulating the size of dendritic spines as well as of INS located on the dendrites and somata of dCA1 PNs by decreasing the size of bigger and increasing the size of smaller synapses at the time scale of days." Is this regulation, or regression to the mean in your analysis? (The largest synapses are simply more likely to become smaller and the smallest ones to become larger).

We frankly do not have enough evidence to answer this question, *i.e.* we cannot exclude that the phenomenon we observed was just due to the fact that "*The largest synapses are simply more likely to become smaller and the smallest ones to become larger*" . Thus, given the marginality of that observation in the economy

of the paper, we decided to eliminate these results from the revised version of the manuscript. We thank Reviewer #3 for asking this question as it has improved our manuscript by eliminating distracting elements and thus sharpening the main message.

4. Page 10: Discussion "Turnover of basal dendritic INS increased immediately after TFC and remained high for several days, leading to their destabilization. This is consistent with previous work in the motor cortex 25". This is ref. 27. It is also consistent with inhibitory synapse destabilization in visual cortex upon plasticity induction (refs 25 and 26).

Thank you for catching that, it is fixed in the revised version.

5. Page 13: Methods "Mice were image using either a Bruker Ultima IV microscope - equipped with an InSight Deep See dual color laser system (Spectra-Physics)" image->imaged

Thank you for catching that, it is fixed in the revised version.

6. Please extend the legend of Fig. 8 so it can be understood without having to go back to the results section.

We have extended the legend of figure 8 as per Reviewer's request.

Klimmt et al.*, NCOMMS-25-22285A - *Answers to Reviewer's commnents

<u>Reviewer #1 (Remarks to the Author):</u>
The authors have responded very adequately to my comments and in my view did so too with respect to the comments and suggestions of the other 2 reviewers. I have no further comments.

We are glad we were able to satisfy Reviewer#1 and we thank them for their support.

<u>Reviewer #2 (Remarks to the Author):</u>
The revisions to the manuscript have largely improved the clarity and confidence of the reported findings. The reformatting of the graphs have also made the revised manuscript easier to follow and understand.
Response to major comments
1. Statistical analysis: The authors have comprehensively updated their data analysis approach to account for grouping of measurements within animals/cells. The analysis now incorporates mixed-effects models with nested variables to avoid treating all data points as independent. Authors provided good justification for use of frequentist statistics (p-values and confidence intervals) for more simple measurements such as size, and Bayesian models in more complex longitudinal measurements such as synaptic survival. The robust analysis approach in the revised manuscripts provides more confidence in the reported results.
2. Excitatory Vs Inhibitory labelling: The authors have removed the direct comparisons between GFP Vs Gephyrin-labelled spines (formerly Fig 5a-b) in acknowledgement of the difference in labelling approaches and have re-phrased discussion to refer to spines rather than excitatory synapses. The authors state they are setting up but not yet practicing equivalent an GFP-Shank labelling approach for excitatory synapses as requested. While not fully addressing the concerns, the revised figure does now show that the difference in putative excitatory and inhibitory "synapse" survival is only evident in newborn and not pre-existing dendrites/INS, which does support the equivalency of this labelling techniques in measuring survival dynamics
3. AIS synaptic clusters: The authors have updated their description of in vivo AIS identification criteria applied and clarified the ex vivo confirmation approach. The limitations of direct comparisons between the stability of single synapses against synaptic clusters (i.e. somatic INS Vs AIS INS) was not resolved, and these comparisons remain as a major component of figure 3. However, the putative influence of synapse clustering on apparent survival was more fully acknowledged in the revised discussion.
4. Discussion: The revised section has been improved with more specific details about proposed contexts e.g. IN population that could explain the result found. Interpretation of data is clearer as a result.
5. Computational modelling: Thank you for clarifying the modelling component.

Response to minor comments
1. AIS identification: Addressed
2. Fig 2e/5a data points: Authors have updated analysis to now look a relative size (ranked) across days (e.g rank order day 1 vs day 2) as shown in figures 2e and 4e. My understanding is that data has been split into subgroups based on how many INS are present on a given neuron. In my opinion, this graph is still a little unclear for example the difference in relative line thickness used to different low vs mid vs high INS containing neurons is very hard to see on the plotted graph. It is also unclear why this subgroup split is included in the plot as I could not find reference to it in the results? Additionally if each line represents a neuron, please include n number somewhere in graph or figure legend.
3. Sampling Frequency: Addressed
4. Fig 3 correlation: Addressed (panel removed)
-Fig 3 data points: Addressed (panel removed)
-Fig 3, panel d: Addressed (panel removed)
-Survival plots: Addressed (Reformatted and mean survival time plot added)
5. Spine Distribution: Addressed
6. Surgical methods: Addressed

New comments
Fig 2d. and 4c What are the empty rows? If an INS isn't present in any of the 13 days (as suggested by empty rows) why is it included?

We are glad we were able to satisfy Reviewer#2 and we thank them for their support.

Regarding *Response to minor comments #2*. While the original legend could give the impression that neurons were formally divided into subgroups according to the number of inhibitory synapses, this was not the intention. Differences in line thickness were included as a visual indication of how many inhibitory synapses, contributed to individual Kendall's τ estimates, not to define or compare separate low, medium-, or high-inhibitory synapses neuron classes. To avoid this misunderstanding, we removed the in-panel legend, and made line thicknesses identical between compartment-specific subpanels. We also added the number of neurons in the figure captions (see lines 979 and 1019).

Regarding *New comments.* There are actually no empty rows within a single animal (we have carefully double-checked the graph at higher magnification). The visual illusion of an empty row is due to the fact that all rows do not always contain a synapse from Day1 and some consecutive rows only contain synapses (or even a single synapse) appearing later on during the experiement. The small thickness of these lines (single synapses) and their (temporal) sparseness gives the false sense of an empty row. Should the Reviewer refer to the empty space between animals, we added a thin line to demarcate different mice.

Reviewer #3 (Remarks to the Author):
I co-reviewed this manuscript with one of the reviewers who provided the listed reports. This is part of the Nature Communications initiative to facilitate training in peer review and to provide appropriate recognition for Early Career Researchers who co-review manuscripts.

Reviewer #4 (Remarks to the Author):
I co-reviewed this manuscript with one of the reviewers who provided the listed reports. This is part of the Nature Communications initiative to facilitate training in peer review and to provide appropriate recognition for Early Career Researchers who co-review manuscripts.

Reviewer #5 (Remarks to the Author):
The authors have addressed most of my concerns with additional experiments and analyses, which I appreciate. However, I remain somewhat unconvinced by the VGAT staining as evidence that somatic Gephyrin-GFP puncta are not aggregates. Colocalization was assessed overall, but not specifically for somatic puncta, and given the high density of VGAT labeling, colocalization may be overestimated. Including a shifted-channel control would help establish chance levels, and this should be feasible with the existing images. I would therefore encourage the authors to assess colocalization specifically for somatic puncta. If these puncta do represent aggregates, it may be more appropriate to exclude those data to avoid potential misinterpretation. While the current caveat in the results section is helpful, resolving this point more directly would strengthen the manuscript. This is not a reason for rejection, but a strong recommendation.

We are glad we were able to satisfy Reviewer#5 and we thank them for their support.

To address the concern that dense VGAT labeling might inflate apparent colocalization, we re-analyzed Gephyrin-GFP puncta separately in the somatic as well as in the dendritic layers using shifted-channel controls on three Gephyrin-VGAT co-labelled images, as *per* Reviewer's suggestion. For each punctum, we defined a local VGAT apposition score as the 95th percentile of VGAT intensity measured within the corresponding Gephyrin-GFP mask. This observed score was then compared to scores obtained after 100 repeated random X-Y shifts of the VGAT channel within the same z-plane. We deliberately used local shifts between 2–3 μm which are sufficient to disrupt true pre/postsynaptic apposition while largely preserving the immediate somatic microenvironment. The rank distributions for somatic puncta were strongly shifted above the chance level of 0.5, with high median ranks and a large fraction of puncta exceeding chance levels (see figure in this paragraph). Thus, the majority of somatic Gephyrin-GFP puncta showed greater local VGAT association than expected from random alignment, arguing against the interpretation

that these puncta primarily represent nonspecific aggregates that are only colocalized with VGAT by chance.

On a final note, we purposely used a N-terminally – not C-terminally - fused construct, which has been shown to increase the synaptic localization in other synaptic labelling approaches (*A Viral Toolbox of Genetically Encoded Fluorescent Synaptic Tags.* Bensussen S, Shankar S, Ching KH, Zemel D, Ta TL, Mount RA, Shroff SN, Gritton HJ, Fabris P, Vanbenschoten H, Beck C, Man HY, Han X.iScience. 2020 Jul 24;23(7):101330. doi: 10.1016/j.isci.2020.101330).

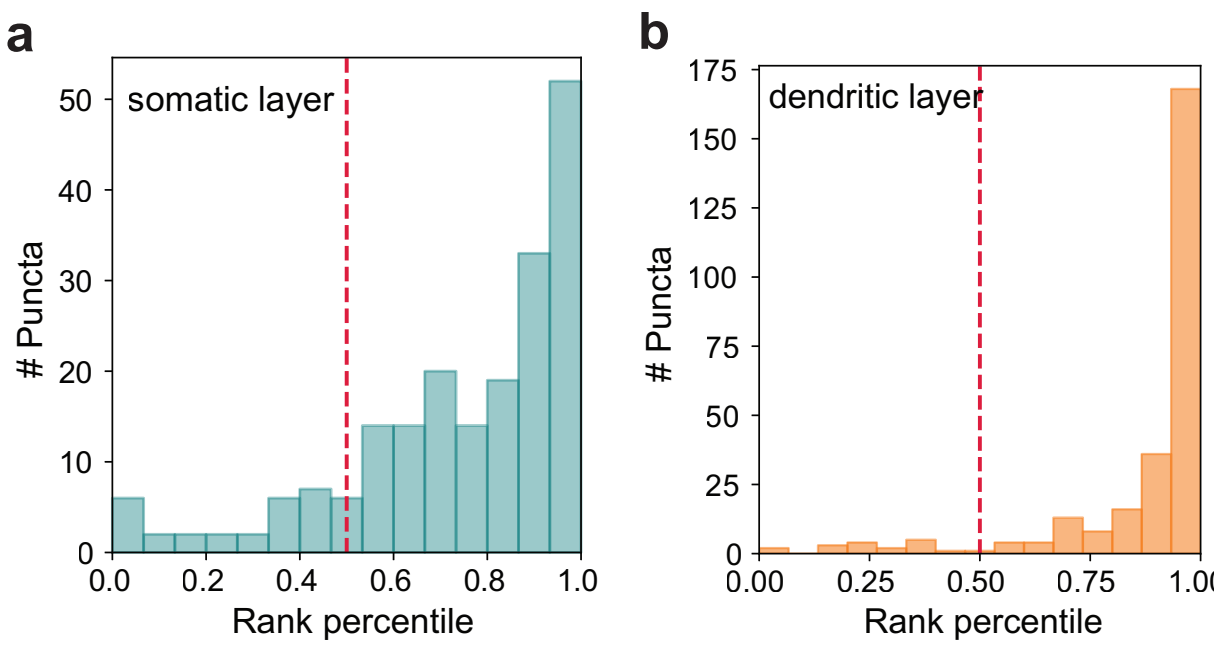

a, b Gephyrin puncta of the somatic layer were reanalyzed using a local shifted-channel control to estimate chance levels of VGAT association. For each punctum, the observed VGAT apposition score was defined as the 95th percentile of VGAT intensity within the corresponding Gephyrin mask and was ranked relative to 100 randomly shifted-channel controls generated by 2–3 µm x-y displacements. Rank distributions for somatic (a) and dendritic (b) inhibitory synapses were shifted clearly above chance level, indicated by the red dashed line (median $\text{rank}_{\text{dendritic}}$ = 0.97, median $\text{rank}_{\text{somatic}}$ = 0.82; $n_{\text{dendritic}}$ = 267, n_{somatic} = 199 puncta).

Finally, "pyramidal neurons" and "PNs" are used throughout the text. Please use "PNs" after introducing the abbreviation.

We thank Reviewer #5 for catching this inconsistency, we have revised to use abbreviations consistently.
